# *Wolbachia* Variants Induce Differential Protection to Viruses in *Drosophila melanogaster*: A Phenotypic and Phylogenomic Analysis

**DOI:** 10.1371/journal.pgen.1003896

**Published:** 2013-12-12

**Authors:** Ewa Chrostek, Marta S. P. Marialva, Sara S. Esteves, Lucy A. Weinert, Julien Martinez, Francis M. Jiggins, Luis Teixeira

**Affiliations:** 1 Instituto Gulbenkian de Ciência, Oeiras, Portugal; 2 Department of Veterinary Medicine, University of Cambridge, Cambridge, United Kingdom; 3 Department of Genetics, University of Cambridge, Cambridge, United Kingdom; Fred Hutchinson Cancer Research Center, United States of America

## Abstract

*Wolbachia* are intracellular bacterial symbionts that are able to protect various insect hosts from viral infections. This tripartite interaction was initially described in *Drosophila melanogaster* carrying wMel, its natural *Wolbachia* strain. wMel has been shown to be genetically polymorphic and there has been a recent change in variant frequencies in natural populations. We have compared the antiviral protection conferred by different wMel variants, their titres and influence on host longevity, in a genetically identical *D. melanogaster* host. The phenotypes cluster the variants into two groups — wMelCS-like and wMel-like. wMelCS-like variants give stronger protection against *Drosophila* C virus and Flock House virus, reach higher titres and often shorten the host lifespan. We have sequenced and assembled the genomes of these *Wolbachia*, and shown that the two phenotypic groups are two monophyletic groups. We have also analysed a virulent and over-replicating variant, wMelPop, which protects *D. melanogaster* even better than the closely related wMelCS. We have found that a ∼21 kb region of the genome, encoding eight genes, is amplified seven times in wMelPop and may be the cause of its phenotypes. Our results indicate that the more protective wMelCS-like variants, which sometimes have a cost, were replaced by the less protective but more benign wMel-like variants. This has resulted in a recent reduction in virus resistance in *D. melanogaster* in natural populations worldwide. Our work helps to understand the natural variation in wMel and its evolutionary dynamics, and inform the use of *Wolbachia* in arthropod-borne disease control.

## Author Summary

*Wolbachia* are bacterial symbionts that infect many arthropods and can protect insects from viral infection. Here we show that different variants of *Wolbachia* from *Drosophila melanogaster* (wMel) are phenotypically heterogeneous: they differ in the level of protection they confer and the titres they reach in their host. The *Wolbachia* with higher titres have higher antiviral protection but can also exert a cost on their host. Based on the observed phenotypes, we divided the wMel variants into two groups and demonstrated that the division is reflected in their phylogeny. Moreover, we discovered the genetic difference between two otherwise almost identical wMel variants, wMelPop and wMelCS, that may explain why one is highly pathogenic while the other produces benign infections. Our study helps to explain the prevalence of the different wMel variants in wild *Drosophila* populations and sheds light on the factors shaping it. In particular, the recent replacement of some wMel variants caused a decrease in anti-viral resistance and probably reduced the cost of the symbiont for the host. Finally, our work helps to understand the interaction of wMel with its natural host and inform *Wolbachia* use in the control of diseases transmitted by insects.

## Introduction

Many arthropods are infected by bacterial secondary (facultative) symbionts [1]. These are vertically transmitted bacteria that are not essential for the host to survive or reproduce, but nonetheless can have important effects on their host's biology. The fitness of these secondary symbionts is directly linked to their host's fitness; their transmission through successive generations is dependent on the breeding success of their hosts. This close association and dependence is predicted to favour the evolution of mutualism [2]. Nonetheless, the presence of replicating bacteria in the host is bound to have a cost. This fitness cost and imperfect vertical transmission would theoretically lead to elimination of vertically transmitted symbionts from host populations [3], [4]. Specific phenotypes associated with secondary symbionts explain their maintenance. Some secondary symbionts are parasites and manipulate their host reproductive biology [3], [5]. Others are mutualists and confer a fitness advantage to their hosts (e.g. resistance to environmental stress or pathogens) [6]. Genetic variability of the symbiont may impact all these associated phenotypes. Therefore, understanding this genotypic and phenotypic variability is essential to understand facultative symbionts population genetics.

In recent years it has become clear that symbionts can modulate the interactions between hosts and parasites in many taxa [6]–[20]. Insects are no exception to this pattern, and secondary symbionts can play a key role in protecting their hosts against infection or parasitism [6], [7], [9]–[12], [14], [17], [19]. Protection to pathogens may be the fitness advantage that enables these bacteria to invade insect populations. For example, the recent spread of *Spiroplasma* in North American populations of *Drosophila neotestacea* may be a consequence of the protection to nematode parasites conferred by these bacteria [10]. Also, the bacterium *Hamiltonella defensa* increases in frequency in aphid cage populations in the presence of parasitoid wasps, to which it provides protection, but decreases in the absence of it [21]. Presence of a protective symbiont can, therefore, be treated as an heritable, albeit non-Mendelian, condition-dependent beneficial genetic change [6].

The intracellular α-proteobacteria *Wolbachia* protects *Drosophila melanogaster* against viral infections [12], [14]. *Wolbachia* are estimated to infect 40% of arthropod species [22] and are, therefore, some of the most common intracellular bacteria known. Its success may be related to its anti-viral protective effect on natural hosts [12], [14], [23], [24], although this protection is not always observed [23], [25], [26]. Other mechanisms, many involving manipulation of the host reproduction, can also maintain *Wolbachia* in natural populations [27], [28]. The most common manipulation is cytoplasmic incompatibility (CI), which renders the crosses between *Wolbachia* infected males and uninfected females sterile or with low viability, giving a relative fitness advantage to infected females. Nonetheless, even when *Wolbachia* can cause CI, a beneficial effect, like protection to viruses, may contribute to the invasion of a new host [29].

*Wolbachia*-conferred protection against viruses is of particular interest because of potential applications in vector-borne disease control. Mosquitoes infected with *Wolbachia* can be more resistant to human arboviruses [24], [25], [30]–[33] and other human pathogens [30], [34]–[36]. A large effort is being made to use *Aedes aegypti* mosquitoes trans-infected with *Wolbachia* variants from *D. melanogaster* in limiting dengue virus transmission [30], [31]. Pilot releases of these trans-infected mosquitoes have already been conducted successfully [37] and intervention in dengue endemic areas is planned [38].

There can be a great deal of genetic variation in how symbionts modulate host-pathogen interactions. Different *D. simulans* lines infected with different *Wolbachia* strains, for instance, show variation in the protection to viruses [23]. The protection ranges from nearly complete to none, and the combinations showing higher protection have higher levels of the endosymbiont [23]. While these *Wolbachia* strains are distantly related, other studies have found variation within populations of closely related symbionts. For example, *H. defensa* protects aphids from parasitoid wasps only when it carries a lysogenic bacteriophage [39]. Understanding this genetic variation among symbionts may explain the frequency of different variants in natural populations and give insight into the mechanisms underlying the interactions.

In natural populations of *D. melanogaster* there has been a recent replacement of *Wolbachia* variants [40]–[42]. *Wolbachia* is present in most natural populations of *D. melanogaster*, although with variable frequencies of infection [41]–[50]. Only a single strain, wMel, is known to infect *D. melanogaster*, but several closely related genotypes of this strain - *w*Mel, *w*Mel2, *w*Mel3, *w*MelCS and *w*MelCS2 - were defined on the basis of polymorphic genetic markers [40]. The frequencies of these genotypes in isolates from natural populations of *D. melanogaster* have changed during the 20^th^ century. Early isolates have a high proportion of wMelCS type, while the wMel genotype is predominant in late 20^th^ century isolates [40]. This wMel genotype replacement was supported by the analysis of *Wolbachia* genotype-associated mitochondrial DNA haplotypes [41]. More recently the genomes of 179 different wMel variants and 290 associated and non-associated mitochondria were assembled [42]. Their analysis showed that all wMel variants come from a single infection event and the most recent ancestor of all wMel and mitochondria dates to about 8,000 years ago [42]. The low genetic diversity and excessive rare variants in wMel, in the well sampled North American population of the *Drosophila* Genetic Reference Panel [51], are consistent with a recent sweep of wMel variants [42]. However, the wMelCS and wMel types diverged several thousand years ago [42] and the sweep is incomplete, since there are still wMelCS variants in natural populations [41], [42], [48], [50].

Phenotypic differences associated with different wMel variants could explain why their frequencies have changed. CI, despite being weak in *D. melanogaster*, has been shown to vary in level in flies harbouring different wMel genotypes [52], [53]. However, the contribution of host or symbiont genetic variation to these differences is not resolved in these studies. Overall clear phenotypic differences between natural wMel variants are not known.

A wMel variant that clearly induces a particular phenotype is wMelPop [54]. This variant was isolated from a laboratory stock and it is pathogenic: it overproliferates and shortens host lifespan [54]–[56]. In terms of genetic markers wMelPop is indistinguishable from wMelCS [57], however, no wMelCS variant with a similar phenotype has been isolated from the wild. Both wMelPop and wMel genotype have been introduced into *Ae. aegypti* as a strategy to block dengue [30], [31] and they protect differently from viral infection [31], [32]. Because of the potential field application and the pathogenicity of wMelPop it is also important to understand in more detail the phenotypic and genomic differences between wMelPop and other variants.

Here we compare the antiviral protection conferred by different wMel variants, in genetically identical *D. melanogaster* hosts. We show that wMelCS-like confer greater antiviral protection than wMel-like variants, but have higher bacterial densities and can reduce the survival of the flies. Through the assembly of their genomes and phylogenetic analysis we reconstruct the relationship of the strains. We also investigate in detail the phenotypic differences between the closely related wMelCS and wMelPop and propose a genomic basis for them. This analysis strengthens the notion that susceptibility to infectious disease can rapidly evolve due to changes in symbionts found in the host population.

## Results

### Phylogenomic Analysis of wMel Variants

**Figure 1:**
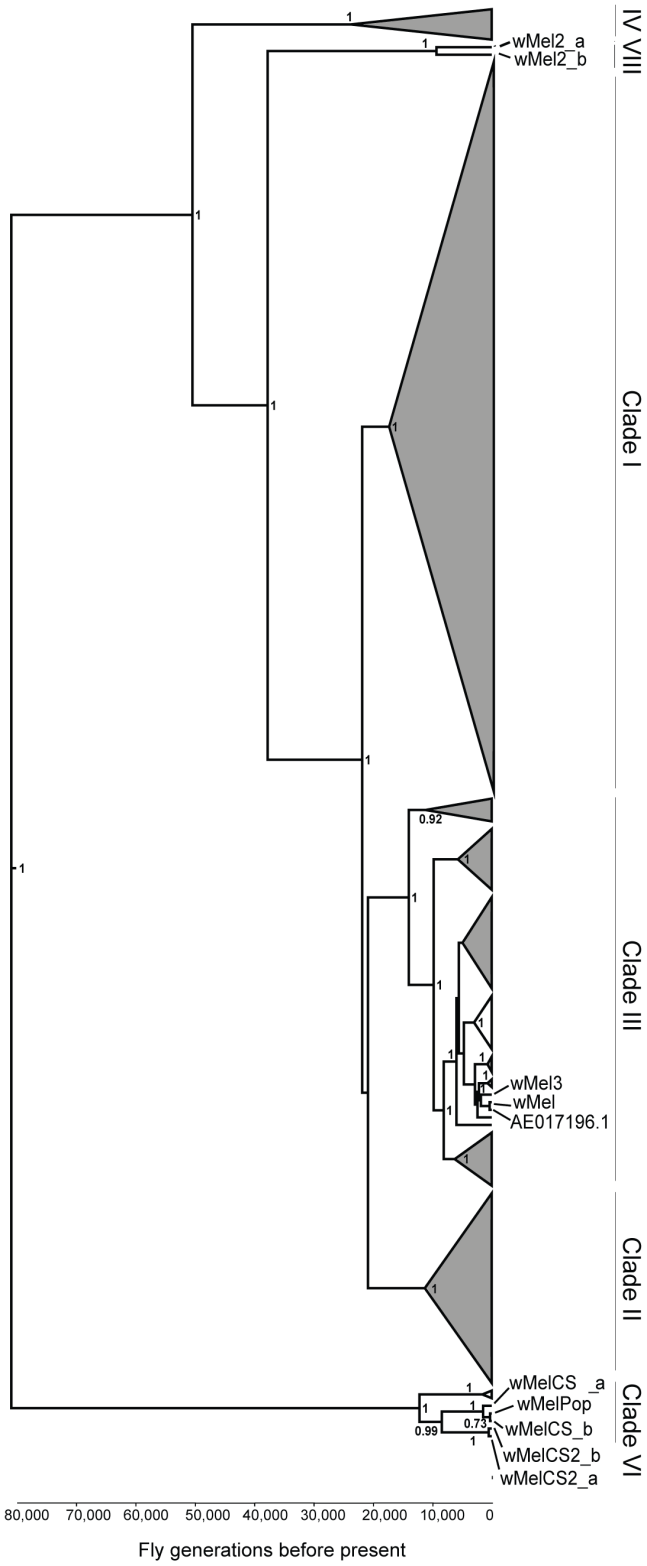
Phylogeny of wMel variants. Phylogenomic tree was reconstructed using the concatenated sequences of complete *Wolbachia* and mitochondrial genomes. The length of the branches reflects the estimated number of *Drosophila* generations, which was calibrated using the mitochondrial mutation rate. The node labels show posterior supports >0.5. The clades are named after Richardson *et al.* [42].

To address the question of how genetic variability within the *Wolbachia w*Mel strain affects resistance to viruses, we analysed the five genotypes described by Riegler *et al.* on the basis of a small number of genetic markers [40]. For the genotypes wMel and wMel3 we used one *D. melanogaster* line, while for wMel2, wMelCS and wMelCS2 we used two lines for each genotype (Table 1). We will refer to each wMel originating from a unique *D. melanogaster* line as a variant. In order to determine the phylogeny of these variants we sequenced and assembled their genomes and associated mitochondria. We sequenced 75 bp paired-end libraries and mapped the reads to the wMel reference genome (GenBank ID: AE017196) and to the mitochondrial genome in *D. melanogaster* Release 5 genome sequence (chrU:5288528–5305749). This mapping strategy was previously used to assemble and analyse the genomes of 179 *Wolbachia* and 290 mitochondria [42]. We produced a phylogenetic tree of the wMel variants together with the 179 *Wolbachia* genomes described in Richardson et *al.* [42] (Figure 1 and Figure S1).

**Table 1:** wMel variants used.

Variant name	*Wolbachia* genotype	Stock name/number	Reference
wMel	wMel	*yw^67C23^*	[40]
wMel3	wMel3	Umea 94/103466	[40]
wMel2_a	wMel2	Amamioshima/E-10032	[40]
wMel2_b	wMel2	Amamioshima/E-10030	[40]
wMelCS_a	wMelCS	Canton S/CS	[40]
wMelCS_b	wMelCS	VF-0058-3	[12]
wMelCS2_a	wMelCS2	Kurdamir/103393	[40]
wMelCS2_b	wMelCS2	Anapa-79/103432	[40]
wMelPop	wMelCS	Popcorn/*w^1118^*	[40]

*Wolbachia* genotypes are based on the diagnostic PCR assays described in Riegler *et al.* [40]. Further information about origin of variant can be found in the indicated reference.

The wMel variant genome clusters together with the reference genome AE017196 in clade III. The only differences we found between them were five positions with an ambiguous call for the wMel nucleotide. This indicates a good quality of the sequencing and assembly, since the reference genome was sequenced from this variant [58]. wMel3 is also assigned to clade III and is the most closely related, out of all the genomes in the phylogenetic tree, to wMel and AE017196. This wMel3 variant is the only known variant with this genotype. The original *D. melanogaster* stock that had wMel3 was probably related to the laboratory stock used for *Wolbachia* wMel sequencing. The only genomic marker from Riegler *et al.* that distinguishes wMel3 from wMel is the absence of the IS5 (ISWip1) WD0516/7 [40]. This seems to be a consequence of a very recent excision of this mobile element in the wMel3 variant since it is present in the closely related wMel variant and in Mel2_a and wMel2_b. Sanger sequencing of this region shows that this would be a precise excision of the transposon (data not shown).

wMel2_a and wMel2_b variants form the new major clade VIII. We estimate that the most recent common ancestor of this clade and clade III dates to 37,537 fly generations before present. The original flies carrying these two variants were captured in the Amami-oshima islands in Japan [40] and eight other lines with wMel2 genotypes have origins in China, Thailand, Philippines and India [41]. Therefore, clade VIII may be exclusive to Asian *D. melanogaster* populations.

wMelCS_a, wMelCS_b, wMelCS2_a and wMelCS2_b variants belong to clade VI and are relatively closely related. As expected from the genomic markers, wMelCS_a and wMelCS_b are more similar to each other than to wMelCS2_a and wMelCS2_b. Our data confirms that wMelCS-like variants belong to clade VI, as predicted by Richardson *et al.*, based on ISWip1 *in silico* mapping [42].

Variants of the genotypes wMel, wMel2 and wMel3 are more closely related to each other than to variants of the wMelCS and wMelCS2 genotypes. We estimate that the most recent common ancestor of all these variants dates back to 80,000 fly generations before present and corresponds to the most recent common ancestor of all wMel variants.

The laboratory variant wMelPop is indistinguishable from wMelCS, based on genomic markers [57]. We have also sequenced and assembled its genome and found it to be closely related to wMelCS_b (Figure 1 and Figure S1).

### wMel Variants Provide Differing Levels of Protection to Viruses

To compare the phenotypic effects of the wMel variants, we replaced the first, second and third chromosome of *Drosophila* lines carrying these variants with chromosomes of the DrosDel *w^1118^* isogenic line [59], using balancer chromosomes. All lines were cleaned of possible chronic viral infections, as previously described [12], [60]. The microbiota associated with these lines, as well as the control *Wolbachia*-free DrosDel *w^1118^* isogenic line (*w^1118^* iso), are expected to be diverse and, presumably, eliminated by the virus cleaning procedure. To homogenize the microbiota associated with these lines, surface sterilized embryos of each line were raised in fly food containing an inoculum of *Drosophila*-associated microbiota from a reference stock.

**Figure 2:**
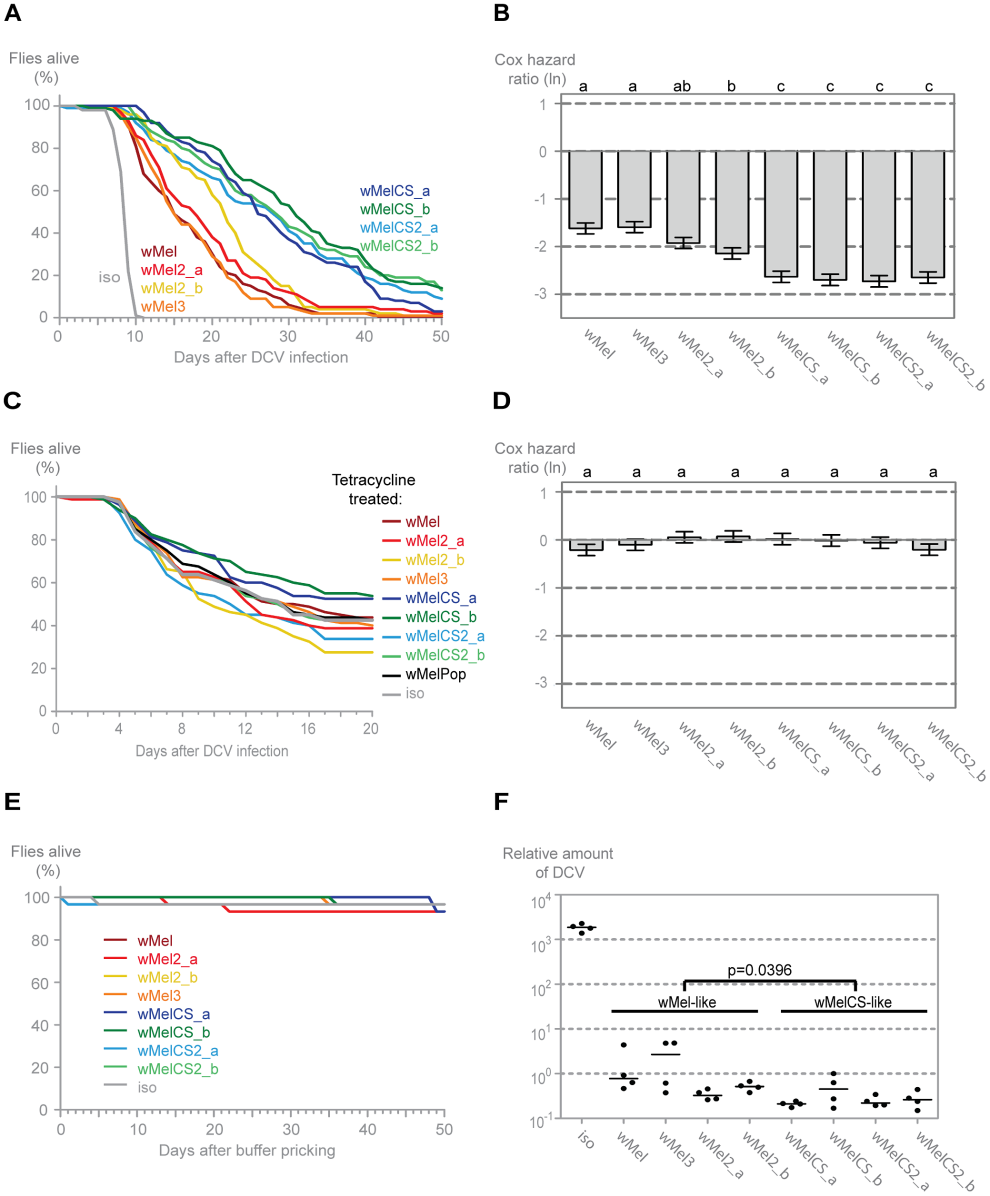
*Wolbachia w*Mel variants confer different protection to *Drosophila* C Virus. (A) One hundred males of each wMel variant line and *w^1118^ iso* were pricked with DCV (10^7.5^ TCID_50_/ml) and survival was followed daily. Two more replicates were performed with similar results. (B) Cox hazard ratio of each wMel variant line compared to *w^1118^ iso* when infected with DCV (10^7.5^ TCID_50_/ml). The natural logarithm of the Cox hazard ratio is shown. Error bars represent standard error. Letters refer to compact letter display of Tukey's test of all pairwise comparisons. Analysis is based on three independent replicates (doses: 10^7^ TCID_50_/ml, 10^7.5^ TCID_50_/ml and 10^9^ TCID_50_/ml), each with 100 flies per line, with 10 flies per vial. *w^1118^ iso* is assigned to group “d” in the compact letter display of Tukey's test (not shown). (C) Eighty males of each tetracycline treated line, derived from the wMel variants lines and *w^1118^ iso*, were pricked with DCV (10^5.5^ TCID_50_/ml) and survival was followed daily. Two more replicates were performed with similar results. (D) Cox hazard ratio of each tetracycline treated line, derived from the wMel variants lines, compared to *w^1118^ iso* tetracycline treated line, when infected with DCV. Analysis is based on three independent replicates, one with 80 flies per line (10^5.5^ TCID_50_/ml) and two with 100 flies per line (one at 10^7^ TCID_50_/ml and one at 10^7.5^ TCID_50_/ml), with 10 flies per vial. *w^1118^ iso* tetracycline treated line is assigned to group “a” in the compact letter display of Tukey's test (not shown). (E) Thirty males of each wMel variant line and *w^1118^ iso* were pricked with buffer and survival was followed daily. (F) 3–6 day old males of each wMel variant line and *w^1118^ iso* were pricked with DCV (10^7.5^ TCID_50_/ml) and collected 3 days later for RNA extraction and RT-qPCR. Relative amount of DCV was calculated using host Rpl32 gene expression as a reference and values are relative to median of wMelCS_b samples. Each point represents a replicate (ten males per replicate, four replicates per *Drosophila* line), and lines are medians of the replicates. DCV loads are two-fold higher in *w*Mel-like variants than in wMelCS-like variants (linear mixed-effect model, *p* = 0.0396).

We tested the mortality after *Drosophila C virus* (DCV) infection in the lines harbouring different *Wolbachia* wMel variants (Figure 2A). DCV is a non-enveloped, positive sense single-stranded RNA virus of the Dicistroviridae family, that is a natural pathogen of *D. melanogaster* [60], [61]. It has been shown before that *Wolbachia* gives strong resistance to this virus [12], [14]. All lines with *Wolbachia* survive the DCV challenge better than the *w^1118^* iso line without *Wolbachia*, demonstrating that all these wMel variants confer protection to DCV. We have analysed the survival data of these infected lines with a Cox proportional hazard mixed effect model [62]. This method determines the Cox hazard ratio for each line, which in this experiment is a measure of the risk of death of DCV-infected flies from each *Wolbachia* line relative to the risk of death of DCV-infected flies from the *Wolbachia*-free line (Figure 2B). A Tukey's test on Cox hazard ratios allows the comparison between all the lines and shows that the *Wolbachia* variants segregate into two groups (Figure 2B). The Cox hazard ratios of the wMelCS-like lines (wMelCS_a, wMelCS_b, wMelCS2_a and wMelCS2_b) are not significantly different from each other but are lower and significantly different from wMel-like lines (wMel, wMel2_a, wMel2_b, and wMel3). Therefore, variants of clade VI (wMelCS-like) confer higher protection to DCV infection than variants of clades III and VIII (wMel-like). There are still some statistically significant differences in survival between lines of the wMel-like group (Figure 2B). However, the statistical analysis does not allow a clear subdivision. In the timeframe of this experiment there is no significant difference in survival between the lines pricked with buffer only (Figure 2E). Therefore, we conclude that the differences in survival upon viral challenge are due to variability in protection to viruses.

*Wolbachia* and mitochondria are both maternally inherited. Consequently, introducing *Wolbachia* variants into the same host genetic background implies co-inheritance of the mitochondria associated with them. To determine the influence mitochondria may have on the survival upon the viral infections we cured all the lines of *Wolbachia* by treating them with tetracycline and we re-homogenized the microbiota in the newly established lines. We have performed this infection with the same dose of DCV as for the *Wolbachia* lines and also with a lower dose in order to better reveal potential differences between lines (all *Wolbachia*-free lines are more susceptible to viral infection) (Figure 2C and 2D). We did not observe any statistically significant difference in survival between the tetracycline-treated lines after DCV infection. A direct comparison between wMel and wMelCS-like derived lines also showed no significant difference (Tukey's test on the mixed effects Cox model fit of the survival data, *p* = 0.953). We conclude that the genetic variability in the mitochondria is not separating these lines regarding the susceptibility to DCV and the original segregation is due to *Wolbachia* variation. However, we cannot formally exclude the possibility of a *Wolbachia*-mitochondria genetic interaction.

To determine if the differences in survival between the two groups were due to differences in viral titres, we assessed the viral load in infected flies using real-time quantitative reverse transcription PCR (qRT-PCR) with DCV-specific primers [63]. We assayed titres at 3 days post-infection, since at this point there is already extensive viral replication but it is not yet at its maximum and there is still no lethality associated with infection [12]. All wMel variants confer resistance to DCV, having on average 5000-fold less virus than control (Figure 2F). The comparison of the virus titres between the wMel-like and wMelCS-like groups shows a significant two-fold difference (linear mixed-effect model, *p* = 0.040). The *Drosophila* lines with wMelCS-like variants have lower viral titres, in agreement with better survival after DCV infection.

**Figure 3:**
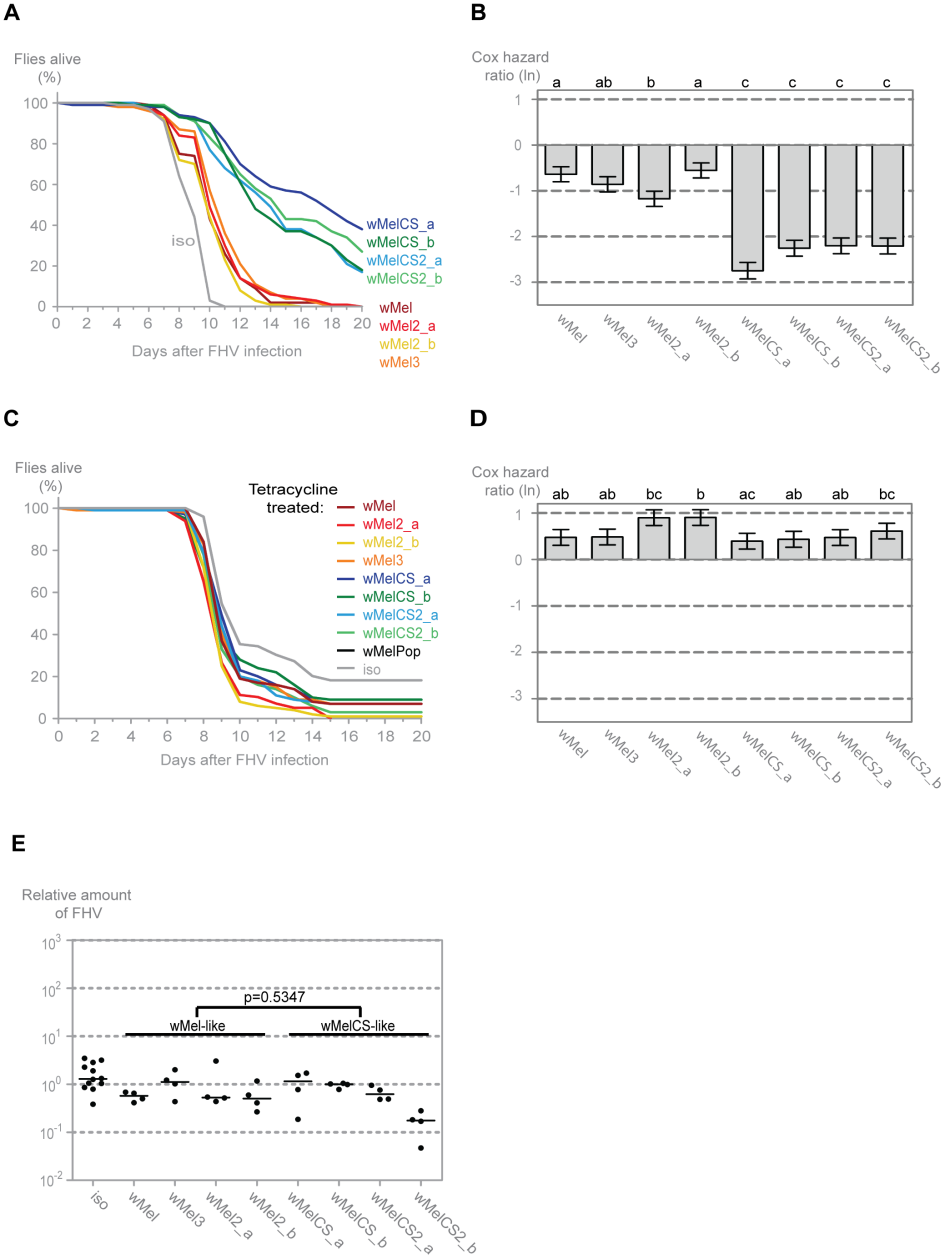
*Wolbachia w*Mel variants confer different protection to Flock house virus. (A) One hundred males of each wMel variant line and *w^1118^ iso* were pricked with FHV (10^9^ TCID_50_/ml) and survival was followed daily. One more replicate was performed with similar results. (B) Cox hazard ratio of each wMel variant line compared to *w^1118^ iso* when infected with FHV (10^9^ TCID_50_/ml). The natural logarithm of the Cox hazard ratio is shown. Error bars represent standard error. Letters refer to compact letter display of Tukey's test of all pairwise comparisons. Analysis is based on two independent replicates, one with 100 flies per line and one with 50 flies per line, with 10 flies per vial. *w^1118^ iso* is assigned to group “d” in the compact letter display of Tukey's test (not shown). (C) One hundred males of each tetracycline treated line, derived from the wMel variants lines and *w^1118^ iso*, were pricked with FHV (10^8^ TCID_50_/ml) and survival was followed daily. (D) Cox hazard ratio of each tetracycline treated line, derived from the wMel variants lines, compared to *w^1118^ iso* tetracycline treated line, when infected with FHV (10^8^ TCID_50_/ml). Analysis is based on one replicate with 100 flies per line, 10 flies per vial. *w^1118^ iso* tetracycline treated line is assigned to group “a” in the compact letter display of Tukey's test (not shown). (E) 3–6 day old males of each wMel variant line and *w^1118^ iso* were pricked with FHV (10^9^ TCID_50_/ml) and collected 3 days later for RNA extraction and RT-qPCR. Relative amount of FHV was calculated using host Rpl32 mRNA as a reference and values are relative to median of wMelCS_b samples. Each point represents a replicate (ten males per replicate, four replicates per *Drosophila* line), and lines are medians of the replicates. FHV loads are not significantly different between *w*Mel and *w*MelCS-like variants (linear mixed-effect model, *p* = 0.5347).

To assess if the wMel variants show differential protection against other viruses we analysed their interaction with Flock House virus (FHV). This is also a non-enveloped positive sense single-stranded RNA virus. However, it belongs to the Nodaviridae family and it is not a natural pathogen of *D. melanogaster* [64], [65]. We have shown before that *Wolbachia* protect *Drosophila* against FHV infections not by limiting the pathogen burden but by increasing survival under similar pathogen load; that is by increasing tolerance to this virus [12], [66]. Consistently with the DCV results we observe that all variants give protection to FHV (Figure 3A). Moreover, the variants split into the same two wMel and wMelCS-like groups, with the latter conferring greater protection (Tukey's test on the mixed effects Cox model fit of the survival data (Figure 3B)). There are, again, some statistically significant differences within the wMel-like group but not between the same variants that show differences in survival after DCV infection.

The survival of the tetracycline treated lines upon infection with a lower dose of FHV showed similar results to the DCV challenge (Figure 3C). Although there are some statistical differences between lines, there is no clear segregation between wMel and wMelCS-like derived lines (Figure 3D) and a direct comparison between these groups showed no significant difference (Tukey's test on the mixed effects Cox model fit of the survival data, *p* = 0.153). This shows that the difference in survival to FHV infection in the non-tetracycline treated lines is not solely due to differences in mitochondria. The differences that we can still observe between lines may be a consequence of differences between mitochondria or due to incomplete isogenization or homogenization of the microbiota in these lines.

To test if there was also a difference in FHV titres between the two groups of wMel variants we measured the levels of this virus three days after infection by qRT-PCR. The comparison of the virus titres between the wMel-like and wMelCS-like groups shows no statistically significant difference (Figure 3E, linear mixed-effect model, *p* = 0.535). Therefore the differences in survival between the two groups are due to differences in tolerance to FHV, not resistance.

The above results show that all tested wMel variants confer protection to DCV and FHV. There is a differential protection that separates the wMel variants into two groups. The wMelCS-like group lines, compared with the wMel-like lines, have a better survival upon infection with both viruses, higher resistance to DCV, and higher tolerance to FHV.

### *Wolbachia* Densities and Host Lifespan Are wMel Variant Dependent

**Figure 4:**
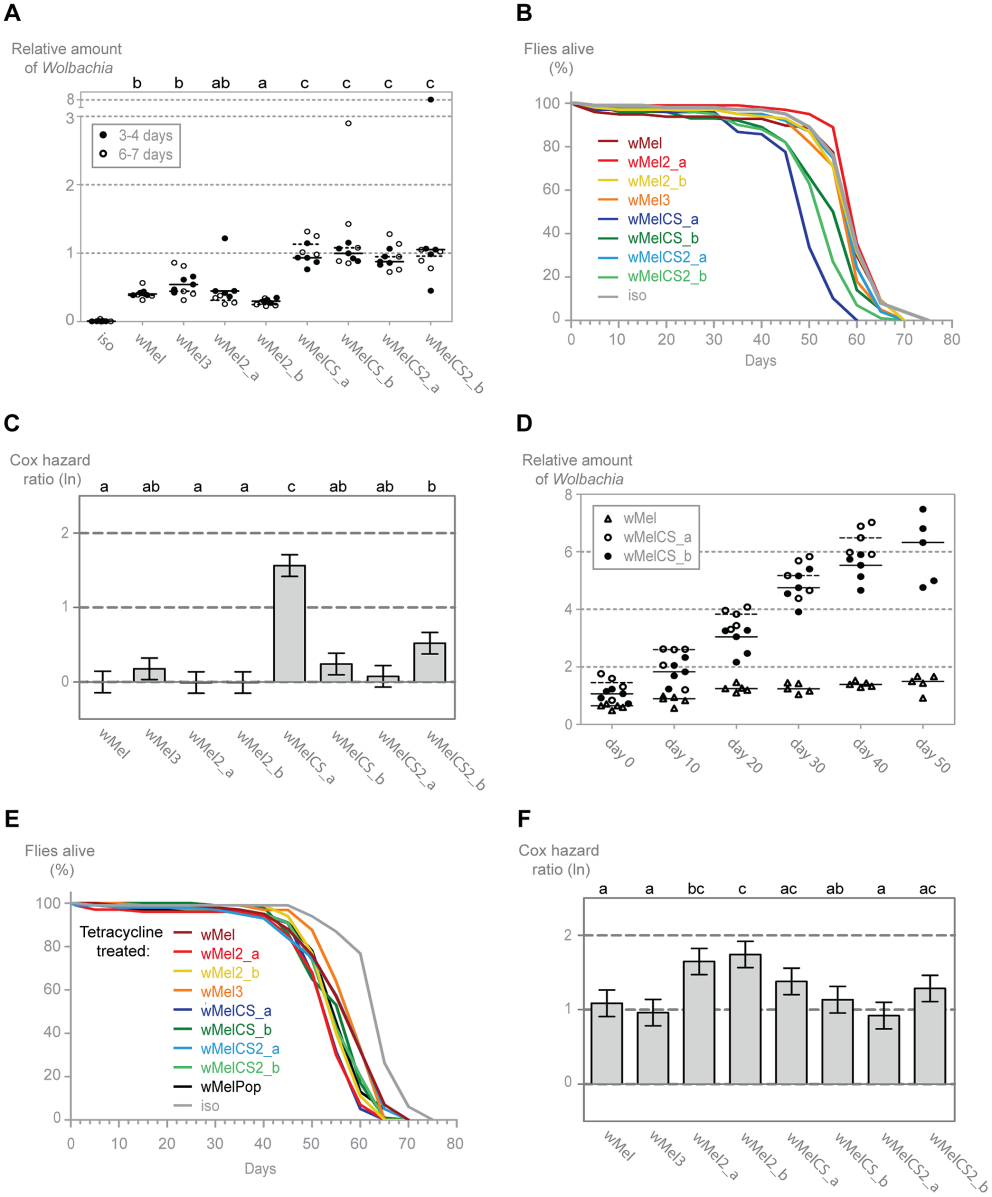
*Wolbachia* densities and *Drosophila* longevity are variant dependent. (A) 3–4 and 6–7 day old males of each wMel variant line and *w^1118^ iso* were collected for DNA extraction and qPCR. Relative amount of *Wolbachia* genomic DNA was calculated using host Rpl32 as a reference gene and values are relative to median of 3–4 days-old wMelCS_b samples. Each point represents a replicate (ten males per replicate, four replicates per *Drosophila* line), and lines are medians of the replicates. The compact letters display of pairwise Wilcoxon rank sum tests between variants is shown on the top. (B) The survival of one hundred males of each wMel variant line and *w^1118^ iso* was checked every five days. One more replicate was performed with similar results. (C) Cox hazard ratio of each wMel variant line compared to *w^1118^ iso*. The natural logarithm of the Cox hazard ratio is shown. Error bars represent standard error. Letters refer to compact letter display of Tukey's test of all pairwise comparisons. Analysis is based on two independent replicates, each with 100 flies per line, with 10 flies per vial. *w^1118^ iso* is assigned to group “a” in the compact letter display of Tukey's test (not shown). (D) Males of wMel, wMelCS_a and wMelCS_b lines were collected for DNA extraction and qPCR every 10 days. Day 0 corresponds to 3–6 days-old flies, wMelCS_a were collected up to 40 days and wMel and wMelCS_b up to 50 days. There are no further time points due to high mortality. Each point represents a replicate (ten males per replicate, five replicates per time point), and lines are medians of the replicates. Relative amount of *Wolbachia* genomic DNA was calculated using host Rpl32 as a reference gene and values are relative to median of samples of wMelCS_b at day zero. (E) The survival of one hundred males of each tetracycline treated line derived from the wMel variants lines and *w^1118^ iso* was checked every five days. The experiment was repeated once with similar results. (F) Cox hazard ratio of each tetracycline treated line, derived from the wMel variants lines, compared to *w^1118^ iso* tetracycline treated line. Analysis is based on two independent replicates, each with 100 flies per line, with 10 flies per vial. *w^1118^ iso* tetracycline treated line is assigned to group “d” in the compact letter display of Tukey's test (not shown).

In order to characterize better the differences between wMel variants and understand the basis of the differential protection, we analysed the titres of *Wolbachia* in the different lines. We determined by qPCR the levels of *Wolbachia* genomes relative to host genomes in males of two age groups: 3–4 and 6–7-day-old. (Figure 4A). We observe that, for each variant, the titre of *Wolbachia* is very similar between the two age groups and that there is no tendency for higher or lower titres at these two time points. However, lines with different wMel variants vary in *Wolbachia* titres and can, once more, be separated into wMelCS and wMel-like groups. A pairwise Wilcoxon rank sum test shows that wMelCS-like variants titres are not significantly different between them but different when compared to wMel-like variants. wMel-like variants show some differences between themselves but with no clear sub-groups. The median *Wolbachia* titre of wMelCS-like lines is 2.55 times higher than wMel-like lines. These results show that wMel titres are, at least partially, controlled by the symbiont genotype.

To determine if these differences in *Wolbachia* titres have any long-term effect on the *D. melanogaster*, we followed the long-term survival of these lines in the absence of any viral challenge (Figures 4B and 4C). The three lines with the shortest average lifespan are all infected with wMelCS-like variants. Of these, the wMelCS_a line has a significantly greater mortality rate compared to all other variants and *w^1118^ iso*, and wMelCS2_b has a statistically significant greater mortality rate than *w^1118^ iso* and three of the wMel-like lines. Despite its shorter mean lifespan, when analysed as proportional hazards the wMelCS_b line is not significantly different from the control. Furthermore, when wMelCS and wMel-like group survivals are directly compared the difference is not significant (Tukey's test on the mixed effects Cox model fit of the survival data, *p* = 0.073). Therefore we can only state that some wMelCS-like variants have a deleterious effect on longevity. Nonetheless, these results exclude the hypothesis that *Drosophila* lines with wMel-like *Wolbachia* succumb to viral infection faster due to a deleterious effect of the variants they are harbouring.

Prompted by these lifespan shortening effects, we investigated how the *Wolbachia* titres of wMelCS_a, wMelCS_b and wMel change through the host life (Figure 4D). We observe that in these three variants *Wolbachia* levels increase with *Drosophila* age (this was not evident in the data set of Figure 4A due to the small interval between the two age groups analysed). Based on the comparison of linear and log-linear models, a linear growth explains better these increases than an exponential one (Table S1). The titres at eclosion are not significantly different between the three variants in a multiple linear regression analysis (intercept wMel: 0.722; intercept difference between wMelCS_a and wMel: 0.416, *p* = 0.090; intercept difference between wMelCS_b and wMel: 0.222, *p* = 0.328). However, while there is a significant increase in wMel titres with the host age (slope wMel: 0.017, *p* = 0.003), the wMelCS_a and wMelCS_b growth rate is 6.5–8 times faster than wMel (slope difference between wMelCS_a and wMel: 0.115, *p*<0.001; slope difference between wMelCS_b and wMel: 0.092, *p*<0.001). wMelCS_a also has a twenty percent faster growth than wMelCS_b (slope difference between wMelCS_a and wMelCS_b: 0.024, *p* = 0.010). These results show that the two tested wMelCS-like variants have a higher growth rate than the wMel-like variant tested. Moreover, wMelCS_a, the variant that shortens host lifespan, has the highest growth rate.

Finally, we analysed the lifespan of the tetracycline treated lines in order to assess the mitochondria contribution to the differences seen in the wMel variants lines survival (Figure 4E and 4F). Although we see small differences between the lines they do not match the differences seen in the wMel variant lines (e.g. the wMelCS_a line treated with tetracycline does not have the shortest lifespan) (Figure 4F). There is no difference in survival of wMel and wMelCS-group derived lines (Tukey's test on the mixed effects Cox model fit of the survival data, *p* = 0.615). We do observe, however, a statistically significant difference between these groups and *w^1118^* iso derived line (*p*<0.001 for wMel-group *vs w^1118^* iso derived lines, *p*<0.001 for wMelCS-group *vs w^1118^* iso derived lines). The *w^1118^* iso line was subjected to the same tetracycline treatment and the difference in survival may be due to variation in mitochondria (see [67]).

Since the wMelCS-like variants have higher titres of *Wolbachia* and better protection to viruses we tested the correlation between *Wolbachia* titres and the survival upon viral infections, viral titres and long-term survival (data in Table S2). We found significant correlations between *Wolbachia* titres and survival upon DCV and FHV infection (Pearson's product moment correlation, *p* = 0.034 and *p* = 0.002 with Bonferroni correction, respectively), but not with the other phenotypes.

**Figure 5:**
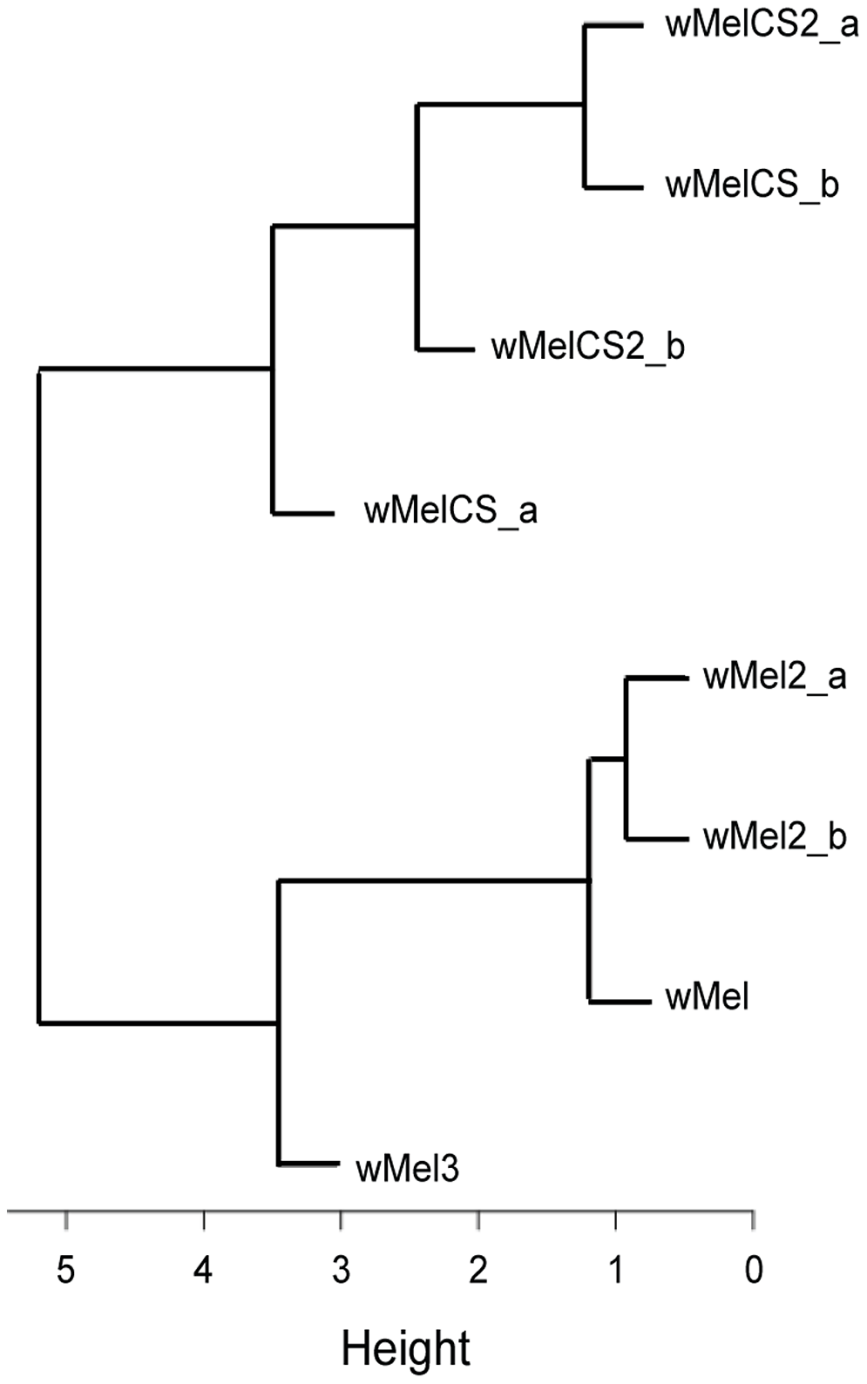
Phenotype-based cluster analysis of wMel variants. Cluster diagram of the wMel variants based on the Euclidian distance of the scaled values of Cox hazard ratios of long-term survival, survival to FVH and DCV infections, FHV and DCV titres upon infection, and *Wolbachia* titres (Data in Table S2).

Given the recurrent phenotypic differences between the wMel-like group and the wMelCS-like group, we tested if, overall, our data led to the clustering of wMel variants into these two groups. To do this we analysed data of survival to viral infection, viral titres upon infections, long-term survival and *Wolbachia* titres together (Figure 5 and Table S2). A cluster analysis of the scaled values, based on Euclidian distances, shows that the wMel variants phenotypes cluster them into a wMel and a wMelCS-like group. This phenotypic clustering (Figure 5) has a phylogenetic basis (Figure 1) and the two groups correspond to the basal clade VI (wMelCS-like) and to variants of the more closely related clades III and VIII (wMel-like).

### *Wolbachia w*MelPop Provides the Strongest Resistance against Viruses

The life shortening wMelPop *Wolbachia* strain is known to over-proliferate in its native *D. melanogaster* host [54] and it has been shown to confer protection to DCV [14]. Importantly, this variant has been transferred to *Aedes aegypti* where it also limits infection by several viruses, like dengue and Chikungunya, and the malaria parasite *Plasmodium gallinaceum* [30], [68]. wMelPop is indistinguishable from wMelCS based on genomic markers [57], therefore we made a detailed comparison between wMelPop and wMelCS_b in the same conditions as for the other wMel variants. However, this set of experiments was performed with 1–2-day-old flies to minimize the variability due to different *Wolbachia* levels within the wMelPop sample or the wMelPop deleterious effect.

**Figure 6:**
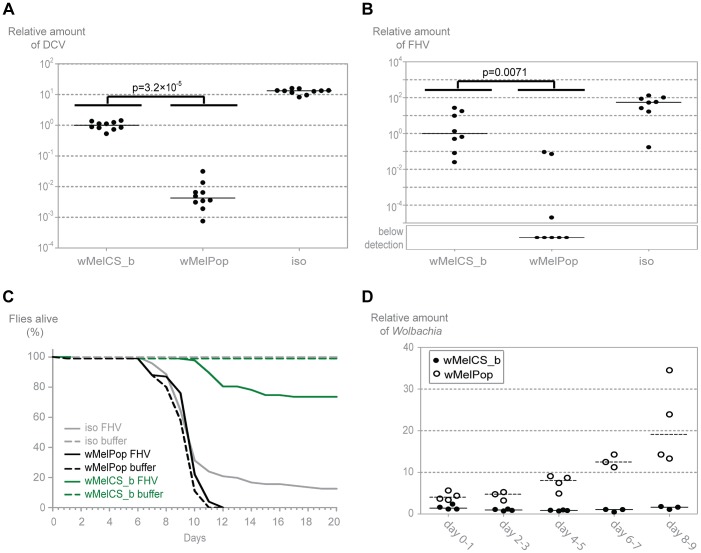
wMelPop confers the strongest antiviral protection. (A) 1–2 day old males of the lines wMelPop, wMelCS_b, and *w^1118^ iso* were pricked with DCV (10^9^ TCID_50_/ml) and collected 3 days later for RNA extraction and RT-qPCR. Relative amount of DCV was calculated using host Rpl32 expression as a reference and values are relative to median of wMelCS_b samples. Each point represents a replicate (ten males per replicate, ten replicates per *Drosophila* line), and lines are medians of the replicates. DCV titres are 235 times lower in wMelPop line than in wMelCS_b line (Mann-Whitney test, *p* = 3.2×10^−5^). (B) 1–2 day old males of the lines wMelCS_b, wMelPop, and *w^1118^ iso* were pricked with FHV (10^7^ TCID_50_/ml) and collected 3 days later for RNA extraction and RT-qPCR. Relative amount of FHV was calculated using host Rpl32 expression as a reference and values are relative to median of wMelCS_b samples. Each point represents a replicate (ten males per replicate, eight replicates per *Drosophila* line), and lines are medians of the replicates. FHV titres are lower in wMelPop line than in wMelCS_b line (Mann-Whitney test, *p* = 0.007). (C) One hundred 1–2 day old males of the lines wMelCS_b, wMelPop, and *w^1118^ iso* were pricked with FHV (10^7^ TCID_50_/ml) or buffer, and the survival was followed daily. (D) Males of wMelCS_b and wMelPop lines were collected for DNA extraction and qPCR every 2 days. Each point represents a replicate (ten males per replicate, three to four replicates per time point), and lines are medians of the replicates. Relative amount of *Wolbachia* genomic DNA was calculated using host Rpl32 as a reference gene and values are relative to median of samples of wMelCS_b at day 2–3.

Upon challenge with DCV, young flies carrying wMelPop have 235-times lower viral loads than the flies with wMelCS_b (over 3000-fold less than *w^1118^* iso) (Figure 6A, Mann-Whitney test, *p*<0.001). wMelPop also has much lower titres of FHV three days post infection when compared with wMelCS_b (Figure 6B, Mann-Whitney test, *p* = 0.007). In most of the wMelPop samples FHV titres were below the limit of detection of the qRT-PCR. Therefore the difference between the medians of wMelCS_b and wMelPop is not quantifiable but it is over ten thousand fold (over one million-fold when compared with *w^1118^* iso). These results show that wMelPop gives stronger resistance to viruses than the closely related wMelCS_b. This data also demonstrates that *Wolbachia* can confer strong resistance to FHV.

We tested wMelPop protection to viral infection in terms of survival upon infection with FHV (Figure 6C). Contrary to wMelCS_b, the presence of wMelPop does not increase survival of FHV infected flies (Tukey's test on the mixed effects Cox model fit, wMelCS_b *versus w^1118^* iso lines infected with FHV, *p*<0.001; wMelPop *versus w^1118^* iso lines infected with FHV, *p = *0.229). This is due to a very strong pathogenic effect of wMelPop, even in the absence of FHV; at 25°C all flies are dead by day 12 (wMelPop *versus w^1118^* iso lines not infected with FHV, *p*<0.001). This pathogenic effect at 25°C has been reported before [54]–[56] but seems stronger in our experiment. Nonetheless, FHV does not cause any mortality in the wMelPop line (wMelPop line infected and not infected with FHV, *p* = 0.816), which is consistent with the strong resistance we observed. Therefore, although wMelPop confers strong resistance to FHV, it does not increase lifespan of an FHV infected host because it is very deleterious by itself.

Given the strong anti-viral resistance and pathogenic effect we observe with wMelPop at 25°C, we decided to measure how *Wolbachia* titres change with age in flies infected with wMelPop and wMelCS_b (Figure 6D). wMelPop growth is better explained by an exponential model of growth than a linear model (Table S1) with an estimated doubling time of 3.4 days. *Wolbachia* titres and growth rate are significantly higher in wMelPop (log-linear model, intercept difference between wMelPop and wMelCS_b: 0.904, *p*<0.001; slope difference: 0.224, *p*<0.001). At the day of our viral infection (1–2 days) wMelPop titres are 3 to 5 times higher than wMelCS_b titres.

Once again we observe that the wMel variant with higher titres gives stronger protection to viruses. In wMelPop the exponential growth leads to a much stronger resistance to DCV and FHV but severely reduces the host lifespan.

### Genetic Basis of the Phenotypic Differences between wMel Variants

Having identified phenotypic differences between wMel variants we asked what their genetic bases were. To answer that used the information from the sequence analysis and their assembled genomes. From the multiple alignments we extracted variant sites that were different between all wMel-like and all wMelCS-like variants, in order to focus on common differences. We detected 108 single nucleotide polymorphisms (SNPs) between these two groups of variants, a tandem duplication and seven insertion-deletion polymorphisms (indels) (Tables 2, S3 and S4). 83 of the SNPs map to annotated wMel genes [58], of which 59 are non-synonymous substitutions. The 55 genes that differ in these 59 SNPs encode proteins with a wide variety of functions, based on predicted conserved domains (Table 2). This set contains a high number of genes coding ankyrin-repeat containing (ANK) proteins: WD0073, WD0292, WD0514, WD0636, WD0754, and WD0766.

**Table 2:** Non-synonymous SNPs between wMel-like and wMelCS-like *Wolbachia* variants.

Gene name		Gene description	Nucleotide			Aminoacid		
			Position	wMel-like	wMel CS-like	Position	wMel-like	wMel CS-like
WD0019		transcription antitermination protein NusG, putative	18552	A	G	191	Q	R
WD0024	rpoBC	DNA-directed RNA polymerase, beta/beta′ subunit	26870	G	A	2060	E	K
WD0033		Piwi/Argonaute/Zwille siRNA-binding domain^a^	36114	C	T	158	V	I
WD0036	prsA	ribose-phosphate pyrophosphokinase	39135	A	C	99	K	Q
WD0041		-	45207	A	G	12	M	T
WD0068		outer membrane protein TolC, putative	65076	A	G	122	N	S
WD0073		ankyrin repeat-containing protein	69287	A	G	298	T	A
WD0086	secD	protein-export membrane protein SecD	79898	G	A	91	T	I
WD0115		transposase, IS4 family	109211	T	G		STOP	E
WD0129		membrane protein CvpA, putative	118051	C	T	15	V	I
WD0130	ribE	riboflavin synthase, alpha subunit	118692	A	G	19	F	S
WD0131		-	119806	A	G	285	L	P
WD0190	mutS	DNA mismatch repair protein MutS	173865	C	T	50	G	R
WD0223		Rossmann-fold NAD(P)(+)-binding proteins; Bacterial NAD-glutamate dehydrogenase^a^	203561	C	T	1233	V	I
WD0223		Rossmann-fold NAD(P)(+)-binding proteins; Bacterial NAD-glutamate dehydrogenase^a^	204413	T	C	949	N	D
WD0262		RuvC_resolvase^a^	248476	G	A	108	A	T
WD0292		prophage LambdaW1, ankyrin repeat domain protein	273138	A	G	40	S	P
WD0363		-	347096	C	T	52	Q	STOP
WD0400		ABC transporter, HlyB/MsbA family, putative	381187	T	C	143	I	T
WD0427	atpB	ATP synthase F0F, A subunit	409057	A	G	139	E	G
WD0433	pccA	propionyl-CoA carboxylase, alpha subunit	414527	G	A	246	T	M
WD0443		OTU-like cysteine protease^a^	427731	C	T	119	R	C
WD0469		cytidine and deoxycytidylate deaminase family protein	452129	C	T	55	S	L
WD0513		RHS repeat-associated core domain^a^	505589	G	A	56	T	I
WD0514		ankyrin repeat-containing protein	506438	C	A	255	A	S
WD0530	pyrH	uridylate kinase	517457	C	T	37	A	T
WD0562		transposase, truncation	547769	C	T	62	E	K
WD0610		helicase, SNF2 family	591593	C	G	126	Q	H
WD0614		O-methyltransferase	598213	G	A	483	D	N
WD0636		ankyrin repeat-containing prophage LambdaW1	628654	G	T	124	A	E
WD0638		Phage tail protein^a^	630778	A	G	112	L	P
WD0639		prophage LambdaW5, baseplate assembly protein J	631303	T	C	201	M	V
WD0666	rplF	ribosomal protein L6	650962	C	T	23	S	N
WD0754		ankyrin repeat-containing protein	728880	T	C	48	E	G
WD0758		glutaredoxin family protein	732864	C	G	18	G	A
WD0766		ankyrin repeat-containing protein	739409	T	G	139	L	W
WD0766		ankyrin repeat-containing protein	739559	T	C	189	I	T
WD0813	proS	prolyl-tRNA synthetase	780933	G	C	196	G	R
WD0814	acpS	holo-(acyl-carrier-protein) synthase	781622	A	G	4	S	G
WD0838		-	803009	G	A	41	V	I
WD0838		-	805011	G	A	709	C	Y
WD0839	uvrB	excinuclease ABC, subunit B	805888	G	C	524	Q	E
WD0867	purH	phosphoribosylaminoimidazolecarboxamide formyltransferase/IMP cyclohydrolase	838894	A	G	260	E	G
WD0898		-	864943	C	A	2	L	F
WD1029	aspC	aspartate aminotransferase	989918	C	G	24	A	G
WD1044		-	1006175	G	A	33	G	D
WD1064	rpoH	heat shock sigma factor RpoH	1024202	A	G	42	N	D
WD1090	rpsA	ribosomal protein S1, putative	1048565	T	C	451	D	G
WD1137		PD-(D/E)XK nuclease family transposase^a^	1089274	T	C	6	I	V
WD1140		PD-(D/E)XK nuclease family transposase^a^	1091606	T	C	34	D	G
WD1200	priA	primosomal protein N	1147604	G	C	423	G	A
WD1216		sensor histidine kinase/response regulator	1164424	C	T	391	H	Y
WD1237	clpA	ATP-dependent Clp protease, ATP-binding subunit ClpA	1185021	G	A	667	A	T
WD1278		-	1220566	A	G	244	Y	C
WD1278		-	1220989	A	G	385	D	G
WD1292		ribonuclease, BN family	1232581	C	T	124	A	V
WD1297		lipolytic enzyme, GDSL family	1239247	C	T	181	R	H
WD1312		DsbA-like disulfide oxidoreductase	1253148	C	T	217	G	E
WD1318	infB	translation initiation factor IF-2	1260309	C	T	309	G	D

Gene name, nucleotide and amino acid positions according to the reference genome AE017196 [58]. Gene description according to the reference genome except in (a), which is based on NCBI CD-search tool [125].

In order to understand the basis of the strong phenotypic differences between the closely related wMelCS_b and wMelPop variants we have investigated the differences between their genomes. Previous studies have not identified any genetic differences between wMelCS and wMelPop [57], [69]. From the genome sequence analysis we found only two SNPs unique to wMelPop, and six positions where there was an ambiguous call for the wMelPop nucleotide. We Sanger sequenced these regions in wMelCS_b and wMelPop, and found that only two synonymous SNPs were true differences between these variants (position 943,443, G>A, unique to wMelPop; position 858,287, T>C, unique to wMelCS_b). In our analysis of split sequencing reads, there were no indel polymorphisms unique to wMelPop that met our filtering criteria. Therefore we cannot identify any SNPs or small indels that could be clearly related to the phenotypic differences.

**Figure 7:**
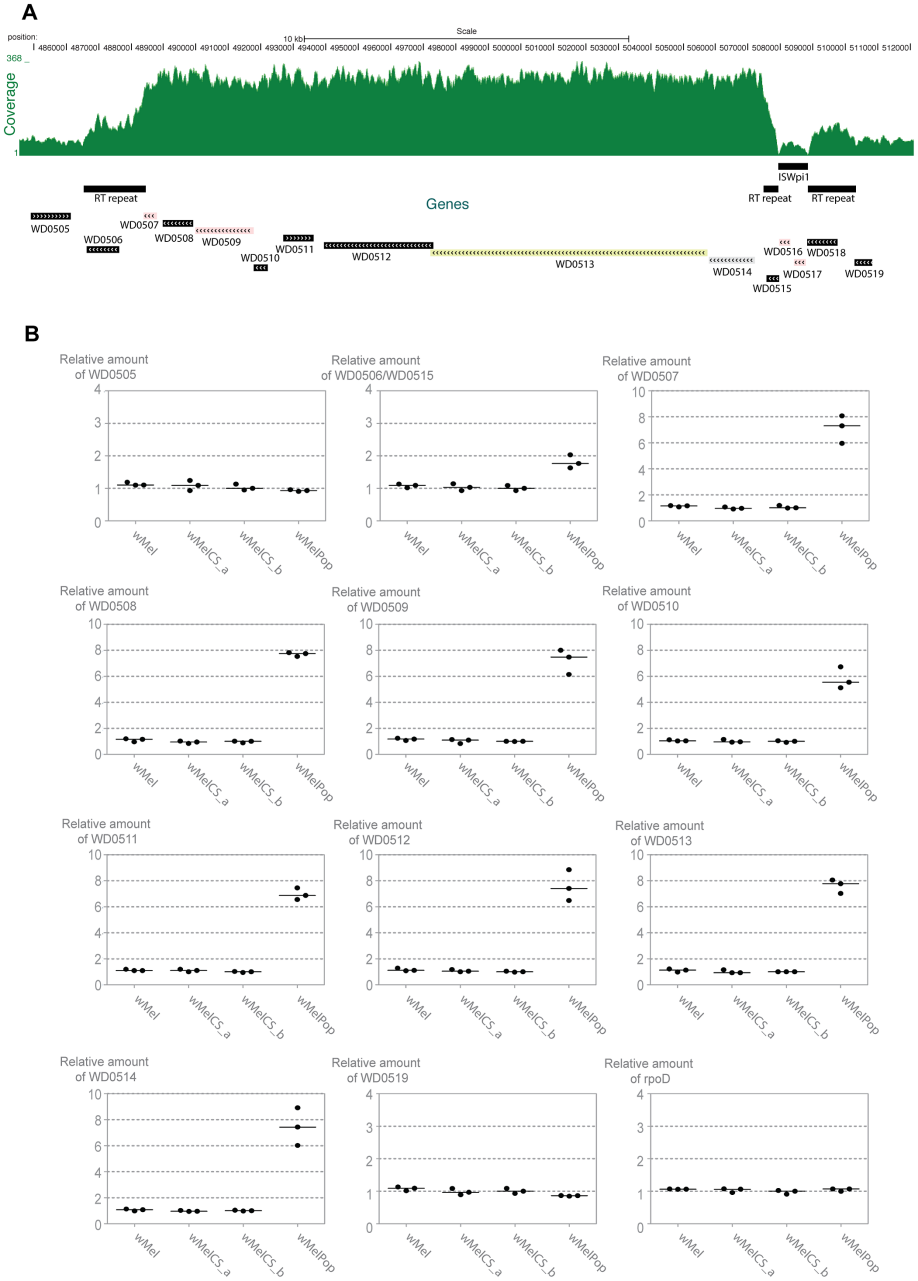
Genomic region amplified in wMelPop. (A) Depth of coverage of sequence reads of wMelPop mapped to wMel reference genome (GenBank: AE017196) in region 484,564 to 512,000. Nucleotide positions, predicted genes, RT repeats and the ISWpi1 element in this region of wMel are shown. The 5′ RT repeat extends from 486,532 to 488,449 (1912 bp). The 3′ RT repeat in wMel extends from 507,470 to 510,325 but is split in two parts due to the insertion of an ISWpi1 (IS5) transposon from 507,928 to 508,848. This ISWpi1 is not present in wMelPop or the closely related wMelCS_b. Figure modified from USCS genome browser (http://genome.ucsc.edu/) [121], [122]. (B) Relative amounts of genomic copy number of Octomom genes (WD0506–14), genes adjacent to Octomom region (WD0505 and WD0519) and control gene *rpoD* in wMel, wMelCS_a, wMelCS_b and wMelPop were calculated using *wsp* as a reference gene. Values are relative to median of wMelCS_b samples. Each point represents a replicate (ten males per replicate, three replicates per *Drosophila* line) and lines are medians of the replicates.

To identify other possible differences between wMelPop and wMelCS_b we analysed copy number variation in their genomes. We mapped the sequence reads to the wMel reference genome and examined variation in the depth of coverage. In wMelPop there is a large increase in read depth in a ∼21 kB region. Using the mean shift approach implemented in CNVnator [70] we estimated that this region has been amplified approximately five times (Figure 7A and Figure S2; t test: *p*<10^−20^; breakpoints: 486,601–507,800). Due to the extensive amplification, probable association with the over-proliferative phenotype, and containing eight predicted genes, we call it the Octomom region.

There are two repeated regions, with the same orientation, flanking the Octomom region (*RT repeat* in Figure 7A). The 5′ repeat region contains WD0506, which is annotated as a pseudogene in the reference genome (GenBank: AE017196 [58]), but it may encode a 329 aa protein with a *reverse transcriptase (RT) with group II intron origin* domain. In the wMel reference genome the 3′ repeat region is split in two parts due to the insertion of an ISWpi1 (IS5) transposon (Figure 7A) (ISWpi1 is repeated 13 times in the wMel genome [58], [71]). This ISWpi1 insertion, however, is absent in the wMelCS-like variants, including wMelPop. In fact presence/absence of this insertion is one of the genomic markers used to distinguish wMel variants (IS5 WD0516/7) [40]. Accordingly, in the coverage plot (Figure 7A) that there is no coverage at the interface between the ISWpi1 and the RT repeat regions in wMelPop. Therefore, this region in wMelPop is 100% identical to the 5′ RT repeat (confirmed, in the region of ISWpi1 insertion, by Sanger sequencing, data not shown). Two other 100% identical RT repeats occur in the genome, at positions 243,822–245,739 and 584,482-582,565 and a smaller 718 bp sequence at positions 633,948–634,665, also 100% identical in its length.

The amplified region in wMelPop contains eight predicted genes between the RT repeats, WD0507–WD0514 (Figure 7A, Table S5). WD0507–11 encode proteins potentially involved in DNA replication, repair, recombination, transposition or transcription. The genes WD0512–14 have previously been shown to be an operon [72]. WD0513 protein has an Rhs domain and WD0514 encodes a ANK repeat protein, but the function of any of the three proteins encoded in this operon is unknown.

The Octomom region was first noticed because of its presence in the strain wMel but absence in many other *Wolbachia* strains [72]. It has since been found that there are homologues of WD0512–14 in wPip [73], [74] and of WD0514 in several strains of *Wolbachia* supergroup A [75]. We find orthologues of all the genes of the Octomom region, including the RT repeat, in the genome of wPip (GenBank: AM999887.1 [76]). In wPip WD0507–10 orthologues have conserved synteny with wMel. We also find WD0507–509 homologue syntenic blocks in the prophages WOVitA1 of wVitA (GenBank: HQ906662.1 [77]) and WOVitB1 of wVitB (GenBank: HQ906666.1 [77])) and in wAlbB (GenBank: CAGB01000117.1).

WD0512–3 and their wPip homologue are also an interesting example of a horizontal gene transfer between *Wolbachia* and mosquitoes [73], [74], [78]. Previously, their homologues have only been found in *Culicidae* (*Aedes*, *Anopheles* and *Culex*). We have also found homologues of WD0513 in the recently sequenced genome of *Daphnia pulex* (GenBank: EFX66732.1 [79]). DAPPUDRAFT_229333 and DAPPUDRAFT_300516 are 35% and 32% identical to this protein, respectively.

To confirm the depth of coverage results we performed qPCR to determine relative genomic copy numbers of the genes immediately adjacent and inside the Octomom region in wMel, wMelCS_a, wMelCS_b and wMelPop (Figure 7B). All genes tested showed the same relative amount in wMel, wMelCS_a, wMelCS_b. The genes immediately outside the Octomom region WD0505 and WD0519, as well as two other control genes located elsewhere in the genome, *rpoD* and *gmk*, show the same copy number in wMelPop and in the other wMel variants (between 0.86 and 1.09 relative to wMelCS_b) (Figure 7B and data not shown). In contrast, in wMelPop the eight genes inside Octomom, WD0507–14, have estimated copy numbers between 5.54 and 7.78 times the levels of wMelCS_b (with a median of 7.42 times). These results confirm the extensive amplification detected by the depth of coverage analysis and show a 7-fold amplification of this region.

The results for WD0506/WD0515 (the qPCR primers amplify both) show 1.77 fold difference between wMelPop and wMelCS_b (Figure 7B). There are 4 identical copies of the amplified region in wMel, wMelCS_a and wMelCS_b (in the 4 full RT repeats). If this region were also amplified 7 times in wMelPop we would expect 2.75 more copies in wMelPop than in wMelCS_b. The fold difference between the wMelPop and wMelCS_b is lower than expected but shows an amplification of this region and indicates that in wMelPop there are 3 more copies of this gene.

The only other large duplication in the wMel variants detected using CNVnator were in wMel2_a and wMel2_b, where a large region corresponding to the phage WO-B has been duplicated (Figure S2; t tests: *p*<0.001; breakpoints in both: 569,001–634,000). This is a stable duplication since the most recent common ancestor of these lines dates to an estimated 9,252 host generations ago (Figure 1). Independent WO-B prophage amplifications have been shown before in *Wolbachia* strains; it is present in two copies in wRi [80] and five copies in wPip [76].

## Discussion

We have found that genetically closely related variants of *Wolbachia* from *D. melanogaster* vary in the degree to which they protect their hosts against viral infection. The *Wolbachia* variants that provide the greatest protection have higher titres and often shorten the lifespan of their hosts (Table S6). Previous work has shown that in natural populations these highly protective wMelCS-like variants were recently largely replaced by less protective wMel-like variants. The genome sequences of strains conferring different levels of protection have allowed us both to reconstruct the evolution of antiviral protection and identify candidate genes that may affect it.

### Phylogeny and Genomics of wMel Variants

Large-scale genome sequencing of wMel variants from natural populations of *D. melanogaster* has previously identified two major monophyletic groups of *Wolbachia* [42]. We were working with a set of *Wolbachia* variants that had been identified using a small number of genetic markers, so we sequenced the genomes of these variants and their associated mitochondria in order to determine where they fall on the phylogeny. We found that these variants belong to both major monophyletic groups, which diverged approximately 80,000 fly generations before present. This date corresponds to the most common recent ancestor of all wMel variants in *D. melanogaster*.

We found that there are striking differences in the degree to which the strains from the different phylogenetic clades protect flies against viruses, with the less common wMelCS-like clade providing the stronger protection and having higher *Wolbachia* densities. This phylogenetic basis for the phenotypic differences confirms that genetic differences between wMel variants are responsible for the variation in symbiont titres and resistance to viruses. Therefore, we identified the common genetic differences between all the wMelCS-like variants and all the wMel-like variants. We found eight indels and 108 SNPs that differ between them, with polymorphisms in the coding sequence of 58 proteins. This number is still too high in order to speculate on possible individual contributions to the phenotypic differences. Future experimental work will help to further reduce this finite number of candidate differences.

We have also compared the genome of the pathogenic variant wMelPop with the closely related wMelCS_b. The wMelPop genome has only two unique differences from the other strains – a single synonymous SNP and a 7-fold amplification of a ∼21 kb region which we named Octomom. The Octomom amplification is therefore the most probable cause of wMelPop pathogenicity and increased protection against viruses. In bacterial genomes copy number variation is very common and mostly involves unequal recombination between two direct sequence repeats, amplifying the region in between [81]. The Octomom region in wMelCS is flanked by two identical 1912b direct repeats, which may provide the origin for the initial duplication in wMelPop. In bacteria and viruses gene amplifications have been shown to increase growth or virulence [81]–[85]. In the future it will be important to show functional data linking this amplification and the pathogenic phenotype of wMelPop.

The functions of the genes in the Octomom region are unknown. The WD0506–WD0511 proteins have predicted domains that are related to interactions with nucleic acids and could have a role in DNA replication, transcription or repair. Therefore, the amplification of these genes could have a direct effect on the replication of *Wolbachia*. WD0512–14 have homology to proteins or protein domains of eukaryotes and are, consequently, candidate effector proteins of *Wolbachia*. Hypothetically they could mediate the pathogenicity through interaction with the host.

Key adaptive traits of bacterial pathogens and symbionts are often controlled by genes that are frequently gained and lost through evolution, which are collectively known as the ‘accessory genome’. The Octomom region appears to fit this pattern as it is partially or totally absent in several *Wolbachia* strains [72], [86], [87]. WD0512–3 homologues have also been suggested to be amplified in wSim [86], although they are not present in its unassembled genome sequence [87]. Homologues of some Octomom genes in other strains have been described [73]–[75], [86] and here we identify more in WOVitA1, WOVitB1, and wAlbB. Moreover, we detect orthologues of all the Octomom genes in wPip, although not as one syntenic block. As the number of sequenced genomes of different *Wolbachia* strains increases it will be interesting to understand the evolutionary history of this region. In particular if there is horizontal gene transfer between strains, as suggested before [72], [74]. This would be compatible with our finding of some of these genes in prophage regions of WOVitA1 and WOVitB1.

The horizontal transfer of these genes may also occur between *Wolbachia* and their insect hosts, as two of the genes in the Octomom region, WD0512–3, are homologous to genes previously identified only in *Culicidae* mosquitoes [73], [74], [78]. The direction of the horizontal gene transfer between mosquitoes and *Wolbachia* is not clear [73], [74], [78]. In mosquitoes these homologues constitute a family of proteins termed salivary gland surface proteins (SGSs). There is evidence that *Ae. aegypti* aaSGS1 is a receptor for malaria sporozoite in salivary glands [78] and *An. gambiae* Sgs4 and Sgs5 are components of the saliva [88]. We have identified two other homologues of WD0513 in the crustacean *Daphnia pulex*. The number of sequenced crustaceans genomes is very low so we do not know how prevalent these genes are in crustaceans. However, the absence of homologues in any other sequenced insect opens the possibility that there was also horizontal gene transfer between *Daphnia*/Crustaceans and either mosquitoes or *Wolbachia*.

### Phenotypes Associated with wMel Variants

Symbionts could protect their hosts against infection either by limiting pathogen titres (resistance) or by reducing the harmful effects of those pathogens (tolerance) [66]. We have previously reported that *Wolbachia* provides tolerance to FHV and resistance to DCV [12]. In this study we found similar FHV titres in lines with wMelCS-like and wMel-like variants, despite the former having far lower mortality rates. This indicates that natural wMel variants differ in how they modulate tolerance to FHV infection rather than resistance (although wMelPop confers strong resistance to FHV, see below). On the other hand, the levels of DCV change between the two groups, with wMelCS-like variants having a two-fold reduction in DCV titres when compared with wMel-like variants. This difference is small, especially when compared to the 5,000-fold reduction in titres in relation to the control without *Wolbachia*, but is reflected in a substantial change in survival. Therefore, it is possible that there is also a tolerance component in the variants differential protection to DCV. However, with our data we cannot distinguish between these hypotheses since it is possible that even a small change in viral titre is sufficient to explain the better survival (see also discussion in [89]). Nonetheless, induced tolerance to DCV has been shown before for the *Wolbachia* strain wRi in *D. simulans* [23]. Therefore, the interaction of *Wolbachia* with different viruses may always have components of resistance and tolerance modulation.

The more protective wMelCS-like variants reach 2.5 higher titres than wMel-like variants in the first days after adult eclosion, and then continue to proliferate during the lifespan of their host. These results show that in *D. melanogaster* the control of *Wolbachia* levels is also dependent on the endosymbiont genotype. It has been shown before that the host genotype and *Wolbachia* strain can influence *Wolbachia* titres [56], [90]–[95]. In *Leptopilina heterotoma* each strain's titre is even independent of the presence of the other strains [93]. Different strains of *Wolbachia* also reach different levels in *D. simulans*, although the host nuclear genetic background has not been controlled in this study [23]. Our results show that these differences are also seen between *Wolbachia* variants that are very closely related to each other (their most recent common ancestor is estimated to date to only 8,000 years or 80,000 fly generations before the present).

The positive correlation between *Wolbachia* titres and protection against viral infection suggests that this may be the cause of the greater protection provided by the wMelCS-like variants. It is important to note that the strains are not phylogenetically independent, so the association between protection to viruses and titres might have arisen independently in the ancestors of the wMel-like and wMelCS-like groups. However, this seems unlikely, as a density effect has been previously reported in *Wolbachia*-mediated antiviral protection. *Wolbachia*-host combinations with higher titres of *Wolbachia* show higher protection [23], [94], [96] and decreasing levels of *Wolbachia* with antibiotic treatment lowers protection [94], [97]. Correlations between titres and other *Wolbachia*-associated phenotypes have been shown before (e.g. with cytoplasmic incompatibility) [95], [98]–[103]. Therefore, the simplest hypothesis is that the differential protection to viruses of the wMel variants is a consequence of their titres.

The localization of the protective symbionts and the pathogens could be an important factor to understand their interaction [23]. *Wolbachia*, DCV and FHV have been shown to infect several tissues of *D. melanogaster* [104]–[108]. Although the information on localizations is not necessarily exhaustive there are some tissues of overlap between *Wolbachia* and the two viruses where the interaction could occur. It will be important in the future to determine the tissue distribution of the different *Wolbachia* variants and how it contributes to the overall differences in titres. It will also be interesting to know if *Wolbachia* titres increase with host age is uniform between all the tissues. It has been previously shown that some *Wolbachia* strains grow at different rates in heads and ovaries [56].

We found that some of the most protective wMel variants reduce the survival of their hosts, suggesting that there may be a trade-off between symbiont-mediated protection and other components of fitness. This cost could be either due to the metabolic cost of their replication or damage caused by their presence. The difference between the wMel-like and wMelCS-like strains was less clear-cut for this trait. We observed that two wMelCS-like lines had significantly greater mortality rates. A third line infected with wMelCS_b has previously been shown to have a shorter lifespan than the control [12], although this is not significant in this report. The fourth wMelCS-like line did not show any detectable effect on lifespan. This variation could be due to the cost of *Wolbachia* infection being difficult to assess in normal laboratory conditions. This reduction in longevity may not directly affect the fitness of flies in the wild since probably not many flies live up to this late age and their fertility would be very low. However, the assay can be interpreted as a proxy for fitness costs associated with the *Wolbachia* variants, which are expressed in other unknown ways in the wild.

The phenotypes of the line carrying the laboratory variant wMelPop are consistent with the differences between natural variants. Our results are in agreement with previous reports that wMelPop can reach high titres and shorten lifespan [54]–[56], as well as to give strong protection to viruses [31], [32]. Here we directly compare this variant with wMelCS_b, its closest related variant, in their natural host. wMelPop is the variant that reaches higher levels in *D. melanogaster*, gives the strongest resistance to viruses, and most severely shortens the host lifespan at 25°C. The pathogenic effect was described before at 25°C [54]–[56]. Yet, this phenotype at 25°C seems to be stronger in our experimental conditions. This is probably related with the wMelPop exponential growth that we detect. We also observed that flies with wMelPop have very strong resistance to DCV and FHV. The strong resistance to FHV induced by wMelPop may indicate that there is no qualitative difference between the interference of *Wolbachia* with DCV and FHV. Again, it may be only a question of different degrees of resistance and tolerance to different viruses.

### Evolution and Dynamics of *Wolbachia* in Populations

Analysis of *Drosophila* lines collected from the early 20^th^ century to the present has indicated that natural selection has driven a recent and fast replacement of wMelCS-like variants by wMel-like variants and their associated mitochondria [40], [41]. A more recent phylogenomic analysis of *Wolbachia* and mitochondria is consistent with a wMel-like global replacement, although it indicates that this event is not complete and started before the 20^th^ century [42]. Overall, it is clear that there was a relatively recent and rapid replacement of wMelCS with wMel-like variants at a worldwide level. Therefore, our results indicate that this has resulted in a recent and rapid decline in the level of anti-viral protection that *Wolbachia* provides *D. melanogaster* in the wild. Consequently, we can conclude that the driving force for this change in wMel frequencies was not an increase in viral protection. On the other hand, the wMelCS-like variants that have higher titres and can have a cost, have been replaced with variants with lower titres and, most probably, lower cost to their hosts.

Our data suggests that the balance between benefit (protection to viruses) and cost may have shifted recently, resulting in selection favouring lower levels of protection. In the simplest scenario, the rate at which this replacement has occurred would allow us to easily estimate the net benefit that the low protection strain has had. There are however several complexities that could affect the dynamics of this replacement. First, if the viruses are predominantly transmitted within *D. melanogaster* populations rather than among different fly species, then the spread of a low protection strain might increase the viral prevalence [29]. This might make the fitness of the low protection strain negatively frequency dependant, potentially stably maintaining both strains in the population. Second, the difference in the density of the high and low protection variants might affect other aspects of *Wolbachia'*s fitness, such as its vertical transmission efficiency or the strength of cytoplasmic incompatibility [95], [103], [109]. These parameters can be experimentally measured and their effects explored with simple extensions to standard models.

In order to block transmission of dengue, the wMel and wMelPop variants were recently introduced into the mosquito *Ae. aegypti* [30], [31]. Our work in *D. melanogaster* is in agreement with the mosquito data showing that wMelPop confers both a higher protection to viruses and a higher fitness cost when compared to wMel [31], [32], [110]. The deployment of these *Wolbachia* infected mosquitoes in the field has to take in consideration the trade-off between fitness costs which make it difficult to invade a population and protection to dengue. Our analysis indicates that wMelCS-like variants have an intermediate phenotype in terms of benefit and cost, and could be considered as an alternative.

Our data also indicate that if there is a strong selection for a mosquito-*Wolbachia* combination with lower fitness costs, this might result in lower protection to viruses. The dynamics of this selection may influence the success of this strategy to control dengue infection. In addition to the replacement of wMelCS-like variants with wMel-like variants in *D. melanogaster*, rapid evolution of *Wolbachia* has been observed in natural populations of *D. simulans*, resulting in an increase in fertility of *Wolbachia* infected flies [111]. Finally, if the Octomom region amplification is the basis of wMelPop higher titres and protection to viruses, it could have important consequences on its long-term maintenance in mosquito populations. Duplications in bacterial genomes can be very unstable due to homologous recombination [81]. If loss of the duplication is frequent in a wMelPop infected mosquito population, a rapid selection of a variant with low replication and low protection to viruses may be expected.

The differences in protection to viral infection with wMel variants demonstrate that in order to understand *Wolbachia* protection to viruses in *D. melanogaster* one has to consider not only presence or absence of *Wolbachia* but also the genetic variability of the symbiont. Our results provide another example of how bacterial symbionts can cause rapid evolution in natural populations and control important traits. Furthermore, they illustrate how the ease with which genomes can be sequenced can provide clues to the molecular basis of these traits.

## Materials and Methods

### Fly Strains and Husbandry

*D. melanogaster* lines with *Wolbachia* are described in Table 1. Lines with *Wolbachia* variants described in Riegler *et al.* [40] were kindly provided by Markus Riegler and Scott O'Neill. wMelCS_b source and DrosDel w^1118^ isogenic background were described elsewhere [12], [59]. wMel variants were introduced in the DrosDel *w^1118^ iso* isogenic background by chromosomes replacement using a first and third double balancer line and a second chromosome balancer line. The crosses were performed with *Wolbachia*-infected females, ensuring endosymbiont transmission through the germline. The fourth chromosome was not isogenized. All the *Wolbachia* genotypes were confirmed by PCR, as described in Riegler *et al.* [40] (data not shown).

The lines were cleaned of possible chronic viral infections as described elsewhere [12], [60].

In order to homogenize the gut microbiota, embryos from each line were sterilized with 2% sodium hypochlorite, followed by 70% ethanol and washed with sterile water. Embryos were placed in new food vials and 150 µl of a bacterial inoculum from a reference stock was added. The inoculum was produced by mixing 5 ml of sterile water with 2 g of food from 10 days old vials containing *VF-0058–3* flies [12], and filtering it to remove eggs and larvae.

Tetracycline-treated lines were cleaned of *Wolbachia* infection by raising them for two generations in ready-mix dried food (Philip Harris) with 0.05 mg/ml of tetracycline hydrochloride (Sigma). Experiments were performed on lines that were raised without antibiotics for at least 6 generations.

*Drosophila* lines were maintained on standard cornmeal diet at a constant temperature of 25°C. We focused the analysis on males in the assumption that *Wolbachia* levels would be more stable in these. *Wolbachia* is present in ovaries and the sizes of these vary greatly with mating status and physiology of the female.

### Long-Term Survival Analysis

To measure the lifespan of different fly lines, 10 flies were placed per vial (without yeast) per replicate, at 25°C. Vials were checked for survival and changed every 5 days.

The analysis of survival data was performed with the Cox proportional hazard mixed effect model. Fixed effects include genotype and repeat of the experiment while replicate vials within the same experiment were considered as a random effect. This method accounts for variation between vials of the same line in the same experiment and variation between replicates of the experiment. Model fitting was done using the coxme package in R [112]. Tukey's test was applied for pairwise comparisons of Cox hazard ratios between all wMel variants and DrosDel *w^1118^ iso.*

### Virus Production and Infection

Viruses were produced and titrated as in Teixeira *et al.* [12], with minor changes. DCV was titrated in Schneider's Line 2 (SL-2), while FHV was titrated in Schneider *Drosophila* line 2 (DL2).

For viral infections CO_2_ anesthetized flies were pricked in the thorax. The 0.15 mm diameter needles used for infection (Austerlitz Insect Pins) were dipped into a virus solution diluted to the desired concentration in 50 mM Tris-HCl, pH 7.5. After the infection flies were kept in vials without yeast, 10 flies per vial. DCV infected flies were maintained at 18°C, while FHV infected flies were maintained at 25°C. Vials were checked for survival daily and changed every 5 days. Unless otherwise stated, infection was performed on 3 to 6 days-old flies.

Survival analysis was done as above.

### RNA Extractions and cDNA Synthesis

For each sample 10 flies were pooled and homogenized with a plastic pestle in 1 ml of Trizol Reagent (Invitrogen). RNA was extracted according to manufacturer's protocol and re-suspended in 50 µl of DEPC-treated water (Ambion). RNA concentrations were determined using NanoDrop ND-1000 Spectrophotometer. cDNA was prepared from 1 µg of total RNA using Random Primers and M-MLV Reverse Transcriptase (both Promega). Primers were allowed to bind to the template RNA for 5 min at 70°C and the reaction proceeded to 25°C for 10 min, 37°C for 60 min and 80°C for 10 min.

### DNA Extractions

For *Wolbachia* relative quantification, ten flies were used per replicate. DNA was extracted according to DrosDel protocol (http://www.drosdel.org.uk/molecular_methods.php) [59]. For wMel Octomom genes relative quantification, total DNA was extracted from replicates of ten flies using a standard phenol-chloroform protocol. The DNA concentrations were checked with NanoDrop ND-1000 Spectrophotometer.

### Real-Time Quantitative PCR

The real-time qPCR reactions were carried out in 7900HT Fast Real-Time PCR System (Applied Biosystems) or CFX384 Real-Time PCR Detection System (BioRad). For each reaction in 384-well plate (Applied Biosystems or BioRad) we used 6 µl of iQ SYBR Green supermix (Bio Rad), 0,5 µl of each primer solution at 3,6 µM and 5 µl of diluted DNA. Each plate contained three technical replicates of every sample for each set of primers. Primers used are described in Table S7.

The thermal cycling protocol for the amplification of *Wolbachia* genes was as follows: initial 50°C for 2 min, denaturation for 10 min at 95°C followed by 40 cycles of 30 s at 95°C, 1 min at 59°C and 30 s s at 72°C. Amplification of DCV and FHV was performed using the same conditions, except an annealing temperature of 56°C. Melting curves were analysed to confirm specificity of amplified products. We obtained Ct values for manual threshold of 10 using the program SDS 2.4 or with Bio-Rad CFX Manager with default threshold settings.

Relative amounts were calculated by the Pfaffl Method [113] using *Drosophila Rpl32* as a reference gene for *wsp* and viruses and *wsp* as a reference for *Wolbachia* Octomom genes.

Kruskal-Wallis rank sum test (kruskal.test in R) was performed on *Wolbachia* and viruses quantification data to detect differences within all the lines. Pairwise comparison between all variants was performed with Wilcoxon rank sum test with Holm correction (pairwise.wilcox.test in R). Direct comparison between wMel-like and wMelCS-like variants was performed with a linear mixed-effects model fit by maximizing the restricted log-likelihood on the log of the values (lme in R). Time course analysis of *Wolbachia* titres was performed with a linear model fit (lm in R).

### Cluster Analysis and Correlations

The data in Table S2 was used for the cluster analysis of wMel variants (hclust in R). In each column the mean was subtracted from the data, for centering, and the result divided by the standard deviation, for scaling. Complete linkage hierarchical clustering was performed on Euclidian distances between wMel variants.

Correlations were calculated using Pearson's product moment correlation (cor.test in R).

### Sequencing and Genome Assembly

The genome assembly of the wMel variants was done with the invaluable help of Casey Bergman (University of Manchester).

For each fly line, 20 females were anaesthetized under CO_2_ and washed in 50% bleach solution for 3 min. Females were then briefly washed in distilled water and dissected under a microscope. The two ovaries of the 20 females were pooled for DNA extraction. DNA was extracted using the Gentra Puregene DNA Purification kit according to the manufacturer's protocol, including an RNase A treatment. Yields of purified DNA ranged between 1.1 and 4.2 µg. Library preparation and sequencing were performed at the Eastern Sequence and Informatics Hub (Cambridge, UK). 75 bp paired-end libraries were prepared with an insert size of 300 bp and sequenced in one lane of HiSeq2000 (Illumina). Base calling was performed using the Offline Basecaller (version 1.9.3) from Illumina, and demultiplexing was handled by bespoke Eastern Sequence and Informatics Hub software. The reads are submitted to the Sequence Read Archive (accession number: ERP002662).

Forward and reverse fastq sequences were mapped individually to single database containing a mitochondrial reference sequence extracted from the *D. melanogaster* Release 5 genome sequence (chrU:5288528–5305749) and the *D. melanogaster Wolbachia* endosymbiont reference genome (GenBank ID: AE017196) and converted to paired end alignments using BWA version 0.5.9-r16 [114]. BWA output was converted to SAM format and reads mapping to the mitochondria or *Wolbachia* reference sequences were extracted and sorted using SAMtools version 0.1.18. Sorted BAM files were used for variant base calling followed by a standard SAMtools version 0.1.16 pileup pipeline [115]. Individual strain consensus fastq sequences were generated using pileup2fq.pl with minimum and maximum read depths set to 10 and 100, respectively, and converted to fasta format using seqtk (https://github.com/lh3/seqtk). Individual reference-based fasta consensus sequence files were merged into multiple alignments from http://bergman.smith.man.ac.uk/data/wolbachia/DGRP_DPGP_Wolbachia_v1.tgz [42]. Alignment columns that had an N in any strain (which can represent either a fully ambiguous character or a deletion relative to the reference) were then removed.

Fasta file of assembled sequences of *Wolbachia* variants and associated mitochondria are in Dataset S1 and S2, respectively. Tables of variants for these *Wolbachia* and mitochondria together with data from *Wolbachia*-carrying strains described in Richardson *et al.* [42] are in Dataset S3 and S4, respectively.

### Phylogenetic Analysis

We produced a dated evolutionary history of *Wolbachia* using BEAST v1.7.2 [116]. The *Wolbachia* and *Drosophila* mitochondrial phylogenies have been shown to be fully congruent [42], so they share the same evolutionary history. We therefore concatenated the *Wolbachia* variants alignments with their respective host *Drosophila* mitochondrial alignments, removing all indels. We included the *Wolbachia* reference strain AE017196, even though no host *Drosophila* mitochondrial alignment exists, after checking that its inclusion made no qualitative difference to either the dates or topology. This alignment was then partitioned into eight different groups representing different categories of sites; first and second codon positions, third codon positions, noncoding RNA genes and intergenic sites (for both the *Wolbachia* and *Drosophila* mitochondria). Each partition had their own HKY+Γ model of evolution [117], [118] but linked to the same dated phylogeny and constant population size coalescent tree prior. In order to calibrate the molecular dating, we assigned a prior lognormal distribution of rate based on the *Drosophila* mutation rate [42], [119] to third codon positions of the *Drosophila* mitochondria, sites that are less likely to be under purifying selection. Rates at all other site classes were given a prior of uniform distribution between 0 and 1, whereas priors on all other parameters were given default values as specified in BEAUti v1.7.2 [116].

### Genetic Polymorphism and Predicted Genes Analyses

For single nucleotide polymorphism analysis a multiple alignment was built, with only the sequences of the wMel variants analysed in this report, and alignment columns that had an N in any strain were removed. Variant sites were then extracted and mapped back to reference coordinates using custom R and PERL scripts (Dataset S5). Variants that differ between all wMel-like and all wMelCS-like variants were identified and mapped to predicted genes or non-coding regions with Galaxy [120]. Identification of synonymous or non-synonymous substitutions was performed with custom Python scripts.

To identify duplications and deletions that have led to copy number variation (CNVs), we examined depth of sequence coverage across the *Wolbachia* genome. To do this we partitioned the genome into non-overlapping 200 bp bins and used the mean shift approach implemented in CNVnator [70] to infer differences in copy number and identify break-points. The variants wMel, wMel3 and wMelCS2_a were not analysed as they had highly variable coverage. Analysis of regions containing duplications was aided by UCSC Genome Browser http://genome.ucsc.edu/ [121], [122].

We also used the program Pindel [123] to search for ‘split reads’, which map to two different positions in the *Wolbachia* genome. To reduce artefacts we only retained structural variants where at least one strain had 10 or more supporting reads and where at least one strain had no supporting reads. As we know the phylogeny of these strains, we expect most true structural variants to be present in monophyletic clades (i.e. they have only arisen once). Out of 18 variants detected, 17 fulfilled this criterion, suggesting that our methods are robust.

Predicted protein domain analysis was based on the reference genome [58] or using NCBI CD-search [124]).

## Supporting Information

Dataset S1 fna file of assembled genomes of wMel, wMel3, wMel2_a, wMel2_b, wMelCS_a, wMelCS_b, wMelCS2_a, wMelCS2_b, and wMelPop.
(ZIP)

Dataset S2 fna file of assembled genomes of mitochondria associated with wMel, wMel3, wMel2_a, wMel2_b, wMelCS_a, wMelCS_b, wMelCS2_a, wMelCS2_b, and wMelPop.
(ZIP)

Dataset S3 Table of variants for new wMel genomes plus Richardson *et al*. [42] wMel genomes.
(TXT)

Dataset S4 Table of variants for new wMel associated mitochondria genomes plus Richardson *et al.* [42] mitochondria genomes associated with *Wolbachia*.
(TXT)

Dataset S5 Table of variants for new wMel genomes only.
(TXT)

Figure S1 Phylogenomic tree of wMel variants analysed. Phylogenomic tree was reconstructed using the concatenated sequences of complete *Wolbachia* and mitochondrial genomes. The length of the branches reflects the estimated number of *Drosophila* generations (shown in the x axis), which was calibrated using the mitochondrial mutation rate. The node labels show posterior supports.
(TIF)

Figure S2 Copy number variation among wMel variants. The black bars represent the number of reads that have been mapped to 200 bp regions of the wMel reference genome. The green line is the estimated copy number relative to the wMel genome. The three large duplications in wMelPop, wMel2_a and wMel2_b were highly significant (t tests: *p*<10^−20^). A smaller duplication in wMelPop was less well supported (t test: *p*<0.03), which is not significant after correcting for multiple tests. wMel, wMel3 and wMelCS2_a were not analysed as they had highly variable coverage.
(TIF)

Table S1 Statistics of linear models based on wMel variants titres change over time. The data for wMel, wMelCS_a and wMelCS_b analysis are represented in figure 4D, wMelPop data are represented in figure 6D.
(DOC)

Table S2 wMel variants phenotypic data for cluster analysis. Natural logarithm of Cox hazard ratios (CHR), relative to *w^1118^ iso*, of survival to infection with DCV and FHV and long-term survival. Median of relative titres of DCV and FHV, three days after infection, and of *Wolbachia*, three and six days after eclosion.
(DOC)

Table S3 Synonymous and non-coding SNPs between wMel-like and wMelCS-like variants. Gene predictions according to annotation of AE017196 [58]. (a) Indicates common ambiguous nucleotide call in the sequence of all wMelCS-like variants (IUPAC nucleotide code).
(DOC)

Table S4 Indels between wMel-like and wMelCS-like variants. a) The type of polymorphism is defined relative to the reference genome AE017196. b) This insertion matches the IS5 insertion in WD1310 described in Riegler *et al.* [40].
(DOC)

Table S5 Predicted genes present in the wMel Octomom region. Gene predictions according to annotation of AE017196 [58]. Domains and predicted functions are based on NCBI CD-Search tool [125]. (a) gene is annotated as a pseudogene, however it contains a valid start site and open reading frame. (b) WD0515 in wMelCS-like variants, including wMelPop, is identical to WD0506.
(DOC)

Table S6 Summary of comparisons between wMel variants phenotypes.
(DOC)

Table S7 Oligonucleotide primers used in real-time quantitative PCR experiments. (a) published in Deddouche *et al.* [63], (b) published in Berry *et al.* [126].
(DOC)
