# Supplementary figures and images for: Wolbachia Variants Induce Differential Protection to Viruses in Drosophila melanogaster: A Phenotypic and Phylogenomic Analysis
Source: PLoS Genet. 2013 Dec 12;9(12):e1003896. doi: 10.1371/journal.pgen.1003896 (PMC3861217; doi:10.1371/journal.pgen.1003896)

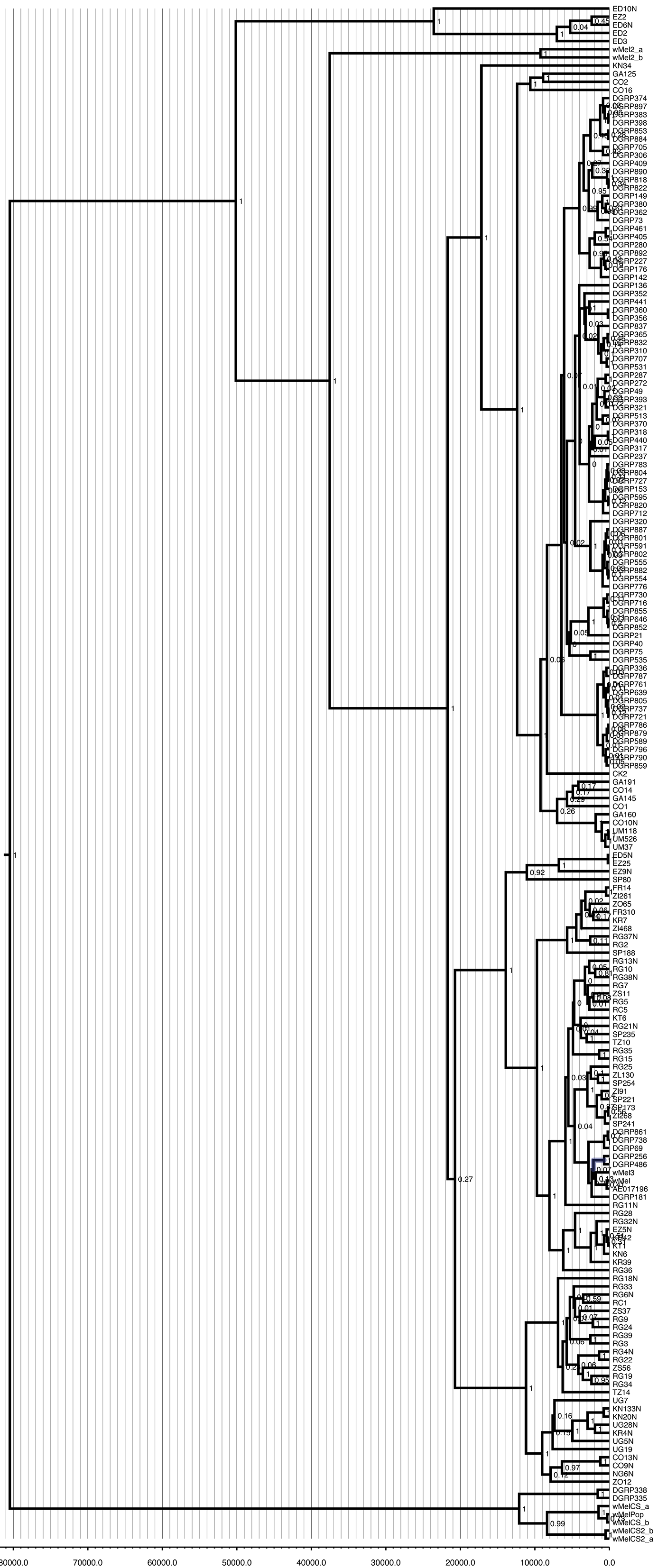

Supplement: Figure S1 — Phylogenomic tree of wMel variants analysed. Phylogenomic tree was reconstructed using the concatenated sequences of complete Wolbachia and mitochondrial genomes. The length of the branches reflects the estimated number of Drosophila generations (shown in the x axis), which was calibrated using the mitochondrial mutation rate. The node labels show posterior supports. (TIF) [file pgen.1003896.s006.tif]

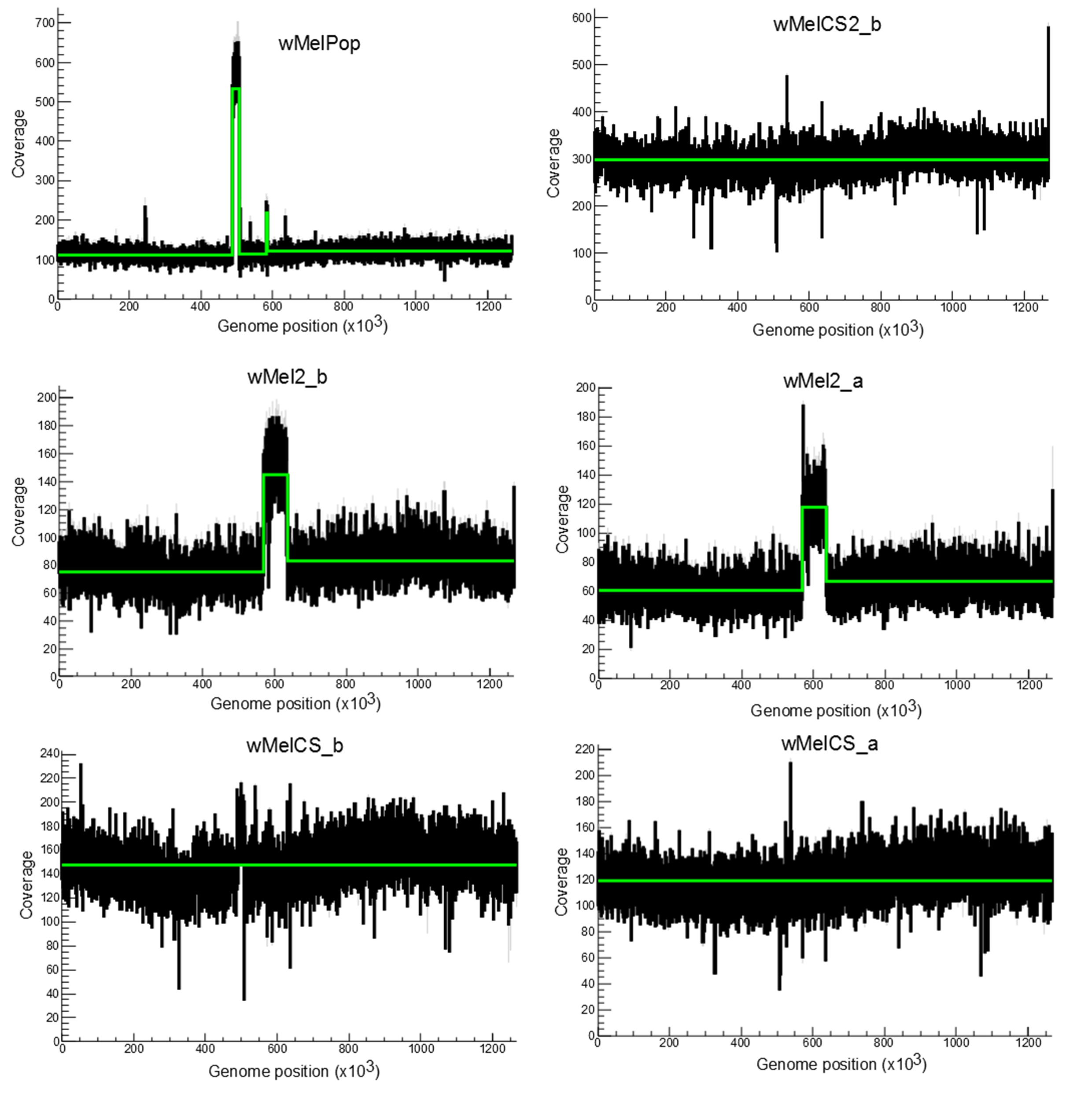

Supplement: Figure S2 — Copy number variation among wMel variants. The black bars represent the number of reads that have been mapped to 200 bp regions of the wMel reference genome. The green line is the estimated copy number relative to the wMel genome. The three large duplications in wMelPop, wMel2_a and wMel2_b were highly significant (t tests: p<10−20). A smaller duplication in wMelPop was less well supported (t test: p<0.03), which is not significant after correcting for multiple tests. wMel, wMel3 and wMelCS2_a were not analysed as they had highly variable coverage. (TIF) [file pgen.1003896.s007.tif]
