# Supplementary material for: Wolbachia Variants Induce Differential Protection to Viruses in Drosophila melanogaster: A Phenotypic and Phylogenomic Analysis
Source: PLoS Genet. 2013 Dec 12;9(12):e1003896. doi: 10.1371/journal.pgen.1003896 (PMC3861217; doi:10.1371/journal.pgen.1003896)
Supplement: Table S1 — Statistics of linear models based on wMel variants titres change over time. The data for wMel, wMelCS_a and wMelCS_b analysis are represented in figure 4D, wMelPop data are represented in figure 6D. (DOC) [file pgen.1003896.s008.doc]

| wMel variant | linear model of titres | | linear model of log of titres | |
| --- | --- | --- | --- | --- |
|  | *R^2^* | *p* | *R^2^* | *p* |
| wMel | 0.6863 | 1.589x10^-8^ | 0.6639 | 4.23x10^-8^ |
| wMelCS_a | 0.934 | 1.801x10^-14^ | 0.8691 | 3.46x10^-11^ |
| wMelCS_b | 0.893 | 4.045 x10^-15^ | 0.8751 | 3.592 x10^-14^ |
| wMelPop | 0.6247 | 9.473 x10^-5^ | 0.8146 | 3 x10^-7^ |
