# Supplementary material for: Wolbachia Variants Induce Differential Protection to Viruses in Drosophila melanogaster: A Phenotypic and Phylogenomic Analysis
Source: PLoS Genet. 2013 Dec 12;9(12):e1003896. doi: 10.1371/journal.pgen.1003896 (PMC3861217; doi:10.1371/journal.pgen.1003896)
Supplement: Table S2 — wMel variants phenotypic data for cluster analysis. Natural logarithm of Cox hazard ratios (CHR), relative to w1118 iso, of survival to infection with DCV and FHV and long-term survival. Median of relative titres of DCV and FHV, three days after infection, and of Wolbachia, three and six days after eclosion. (DOC) [file pgen.1003896.s009.doc]

| Variant | DCV CHR | FHV CHR | Longterm CHR | DCV titres | FHV titres | *Wolbachia* titres |
| --- | --- | --- | --- | --- | --- | --- |
| wMel | -1.619 | -0.637 | -0.001 | 1.880 | 0.603 | 0.393 |
| wMel2_a | -1.925 | -1.175 | -0.008 | 0.787 | 0.557 | 0.380 |
| wMel2_b | -2.145 | -0.554 | -0.008 | 1.252 | 0.527 | 0.288 |
| wMel3 | -1.593 | -0.858 | 0.175 | 6.539 | 1.178 | 0.506 |
| wMelCS_a | -2.633 | -2.748 | 1.564 | 0.514 | 1.212 | 0.980 |
| wMelCS_b | -2.698 | -2.255 | 0.241 | 1.095 | 1.053 | 1.032 |
| wMelCS2_a | -2.728 | -2.203 | 0.075 | 0.535 | 0.655 | 0.912 |
| wMelCS2_b | -2.649 | -2.209 | 0.521 | 0.635 | 0.184 | 1.022 |
