# Supplementary material for: Wolbachia Variants Induce Differential Protection to Viruses in Drosophila melanogaster: A Phenotypic and Phylogenomic Analysis
Source: PLoS Genet. 2013 Dec 12;9(12):e1003896. doi: 10.1371/journal.pgen.1003896 (PMC3861217; doi:10.1371/journal.pgen.1003896)
Supplement: Table S3 — Synonymous and non-coding SNPs between wMel-like and wMelCS-like variants. Gene predictions according to annotation of AE017196 [58]. (a) Indicates common ambiguous nucleotide call in the sequence of all wMelCS-like variants (IUPAC nucleotide code). (DOC) [file pgen.1003896.s010.doc]

| Position | Nucleotide wMel-like | Nucleotide wMelCS-like | Gene name |
| --- | --- | --- | --- |
| 15742 | A | G | WD0016 |
| 25603 | G | A | WD0024 |
| 45292 | C | T | Non-coding region |
| 89970 | A | G | Non-coding region |
| 94978 | T | C | WD0103 |
| 151274 | T | A | Non-coding region |
| 183394 | G | A | Non-coding region |
| 201340 | G | A | WD0221 |
| 278050 | G | A | Non-coding region |
| 287099 | G | A | Non-coding region |
| 346902 | G | A | Non-coding region |
| 372405 | A | G | Non-coding region |
| 398613 | A | G | Non-coding region |
| 416891 | C | T | WD0435 |
| 440973 | A | C | WD0460 |
| 449370 | A | G | Non-coding region |
| 463714 | T | C | Non-coding region |
| 537479^a^ | C | Y | WD0550 |
| 537486^a^ | T | Y | WD0550 |
| 537512^a^ | T | Y | WD0550 |
| 571424 | G | T | Non-coding region |
| 587192 | G | T | WD0609 |
| 588436 | T | C | Non-coding region |
| 654265 | T | C | WD0675 |
| 738991 | A | G | Non-coding region |
| 739429^a^ | A | R | WD0766 |
| 739618^a^ | G | S | WD0766 |
| 811613 | G | A | WD0847 |
| 811613 | G | A | WD0848 |
| 812321 | A | G | Non-coding region |
| 830307 | C | G | Non-coding region |
| 840037 | A | G | Non-coding region |
| 854922 | T | K | Non-coding region |
| 872208 | G | A | Non-coding region |
| 889384 | G | A | WD0924 |
| 914712 | G | C | Non-coding region |
| 917945 | A | G | Non-coding region |
| 949888 | G | A | WD0992 |
| 988727 | C | T | Non-coding region |
| 1017650 | T | C | WD1055 |
| 1135851 | T | C | Non-coding region |
| 1145254 | T | C | WD1199 |
| 1152452 | T | C | WD1203 |
| 1165158 | G | A | WD1217 |
| 1183214 | C | T | WD1237 |
| 1206452 | T | C | Non-coding region |
| 1207767 | G | A | WD1262 |
| 1217973 | G | A | WD1277 |
| 1247609 | A | G | Non-coding region |
