# Supplementary material for: Wolbachia Variants Induce Differential Protection to Viruses in Drosophila melanogaster: A Phenotypic and Phylogenomic Analysis
Source: PLoS Genet. 2013 Dec 12;9(12):e1003896. doi: 10.1371/journal.pgen.1003896 (PMC3861217; doi:10.1371/journal.pgen.1003896)
Supplement: Table S4 — Indels between wMel-like and wMelCS-like variants. a) The type of polymorphism is defined relative to the reference genome AE017196. b) This insertion matches the IS5 insertion in WD1310 described in Riegler et al. [40]. (DOC) [file pgen.1003896.s011.doc]

| Type ^a^ | Size | Inserted sequence | Start | End | Gene name | Protein domains |
| --- | --- | --- | --- | --- | --- | --- |
| long insertion^b^ | unknown | unknown | 1251892 | 1251892 | WD1310 | P-loop NTPase |
| deletion | 6 | none | 45287 | 45294 | Non-coding region | - |
| deletion | 1 | none | 222378 | 222380 | Non-coding region | - |
| deletion | 9 | none | 864708 | 864718 | WD0898 | no predicted domains |
| short insertion | 5 | AGAGT | 156880 | 156881 | Non-coding region | - |
| short insertion | 1 | T | 279146 | 279147 | Non-coding region | - |
| short insertion | 1 | A | 432673 | 432674 | Non-coding region | - |
| tandem duplication | 99 | - | 537421 | 537521 | WD0550 | ankyrin-repeat containing protein |
