# Supplementary material for: Wolbachia Variants Induce Differential Protection to Viruses in Drosophila melanogaster: A Phenotypic and Phylogenomic Analysis
Source: PLoS Genet. 2013 Dec 12;9(12):e1003896. doi: 10.1371/journal.pgen.1003896 (PMC3861217; doi:10.1371/journal.pgen.1003896)
Supplement: Table S5 — Predicted genes present in the wMel Octomom region. Gene predictions according to annotation of AE017196 [58]. Domains and predicted functions are based on NCBI CD-Search tool [125]. (a) gene is annotated as a pseudogene, however it contains a valid start site and open reading frame. (b) WD0515 in wMelCS-like variants, including wMelPop, is identical to WD0506. (DOC) [file pgen.1003896.s012.doc]

| Predicted gene | Size (aa) | Domain / predicted function |
| --- | --- | --- |
| WD0506^a^ | 329 | Reverse transcriptase (RTs) with group II intron origin |
| WD0507 | 135 | RadC domain - DNA repair protein |
| WD0508 | 312 | Helix-turn-helix XRE-family like proteins - DNA binding protein |
| WD0509 /MultL-2 | 598 | MutL - DNA mismatch repair protein |
| WD0510^a^ | 146 | RNase HI prokaryote like |
| WD0511 | 309 | PD-(D/E)XK nuclease family transposase – putative transposase, DNA invertase (resolvase), or recombinase |
| WD0512 | 1120 | - |
| WD0513 | 2843 | RHS repeat-associated core domain |
| WD0514 | 469 | Ankyrin repeats |
| WD0515^b^ | 329 | Reverse transcriptase (RTs) with group II intron origin |
