# Supplementary material for: Wolbachia Variants Induce Differential Protection to Viruses in Drosophila melanogaster: A Phenotypic and Phylogenomic Analysis
Source: PLoS Genet. 2013 Dec 12;9(12):e1003896. doi: 10.1371/journal.pgen.1003896 (PMC3861217; doi:10.1371/journal.pgen.1003896)
Supplement: Table S6 — Summary of comparisons between wMel variants phenotypes. (DOC) [file pgen.1003896.s013.doc]

| Comparison | DCV infection | FHV infection | *Wolbachia* levels | Lifespan |
| --- | --- | --- | --- | --- |
| wMelCS-like compared to  wMel-like | - Better survival  - 2-fold lower titres  - Higher resistance  - Higher tolerance? | - Better survival  - No difference in titres  - Higher tolerance | - Higher titres  - Higher growth rate | - Some lines have reduced survival |
| wMelPop  compared to wMelCS_b | - Strongly reduced titres  - Higher resistance | - Strongly reduced titres  - Higher resistance | - Higher titres  - Much higher growth rate | - Strongly reduced survival |
