# Supplementary material for: Wolbachia Variants Induce Differential Protection to Viruses in Drosophila melanogaster: A Phenotypic and Phylogenomic Analysis
Source: PLoS Genet. 2013 Dec 12;9(12):e1003896. doi: 10.1371/journal.pgen.1003896 (PMC3861217; doi:10.1371/journal.pgen.1003896)
Supplement: Table S7 — Oligonucleotide primers used in real-time quantitative PCR experiments. (a) published in Deddouche et al. [63], (b) published in Berry et al. [126]. (DOC) [file pgen.1003896.s014.doc]

| Target | Forward primer sequence (5’- 3’) | Reverse primer sequence (5’- 3’) |
| --- | --- | --- |
| DCV^a^ | TCATCGGTATGCACATTGCT | CGCATAACCATGCTCTTCTG |
| FHV^b^ | ACCTCGATGGCAGGGTTT | CTTGAACCATGGCCTTTTG |
| Rpl32 | CCGCTTCAAGGGACAGTATC | CAATCTCCTTGCGCTTCTTG |
| wsp | CATTGGTGTTGGTGTTGGTG | ACCGAAATAACGAGCTCCAG |
| WD0505 | TGTTCCTGGTGGATCATCTG | ACGCGAGCATCTTCCATAAG |
| WD0506/WD0515 | TTTGCGTCTTCTTCCCTCTC | ATCAAGGCACACCACAAGGT |
| WD0507 | GCATGACAGGGAAGAAGCTC | CTTTGCAGCTTCCTTTAGGC |
| WD0508 | TCTAGCTTGCGGACAAGAAG | CTGCCTTTCCACTTTCTTCC |
| WD0509 | CCGTATAGCAGCAGGAGAGG | AGTGGCATGCCTCATAAGTG |
| WD0510 | CCACTTGTTGATCCATCCTG | GGCAGCCGTGGTAATGTATG |
| WD0511 | CTTGGCTGCTATTCACGATG | CGAAGCCCTTGGTCTTAGTG |
| WD0512 | ATGCTGCTAATTGGGACTGG | AGGCAATCGACCATACTTGC |
| WD0513 | TTAACCGGCCAGTCTTATCG | AGCATGTCCTCTCTGCCATC |
| WD0514 | CTGTGCCTGAGAATCAAGAGG | CCTTCAAGCGAGGAGATTTG |
| WD0519 | TGCAAGAAGAGAAAATCAAATAAGAG | TCCCTTGTAAGCGTTCTTTC |
| rpoD | AAGAGGCCTTGATCTGCTTG | CCACCAAGTGCCATAAGTTG |
| Gmk | ACTGGCAAGGAGCATTTCAC | CGCTCTATTTCGCTTGCATC |
